# Harnessing low dimensionality to visualize the antibody–virus landscape for influenza

**DOI:** 10.1038/s43588-022-00375-1

**Published:** 2022-12-30

**Authors:** Tal Einav, Adrian Creanga, Sarah F. Andrews, Adrian B. McDermott, Masaru Kanekiyo

**Affiliations:** 1 Basic Sciences Division and Computational Biology Program, Fred Hutchinson Cancer Research Center, Seattle, WA USA; 2 Vaccine Research Center, National Institute of Allergy and Infectious Diseases, National Institutes of Health, Bethesda, MD USA

**Keywords:** Influenza virus, Antibodies, Computational models

## Abstract

Antibodies constitute a key line of defense against the diverse pathogens we encounter in our lives. Although the interactions between a single antibody and a single virus are routinely characterized in exquisite detail, the inherent tradeoffs between attributes such as potency and breadth remain unclear. Moreover, there is a wide gap between the discrete interactions of single antibodies and the collective behavior of antibody mixtures. Here we develop a form of antigenic cartography called a ‘neutralization landscape’ that visualizes and quantifies antibody–virus interactions for antibodies targeting the influenza hemagglutinin stem. This landscape transforms the potency–breadth tradeoff into a readily solvable geometry problem. With it, we decompose the collective neutralization from multiple antibodies to characterize the composition and functional properties of the stem antibodies within. Looking forward, this framework can leverage the serological assays routinely performed for influenza surveillance to analyze how an individual’s antibody repertoire evolves after vaccination or infection.

## Abstract (web summary)

A framework is presented to extrapolate the range of behaviors for influenza antibodies. Using this basis set of behaviors, the collective action of multiple antibodies can be teased apart to describe the individual antibodies within.

## Main

A key problem in immunology is to discover antibodies that can protect against a wide range of viruses. However, it is difficult to quantify the inherent tradeoff between antibody potency (how well a virus is neutralized) and breadth (how many different viruses are neutralized). This tradeoff is especially important for rapidly evolving viruses such as influenza, where we seek antibodies that are both highly potent and broadly neutralizing^1–3^. Because these goals can be mutually exclusive, and because characterizing new antibodies is time- and resource-intensive, we need a framework that extrapolates the behavior of a few antibodies to describe other phenotypes.

The situation is further complicated in the context of multiple (polyclonal) antibodies, as in our immune system. With every infection or vaccination against the influenza virus, our antibody repertoire is reshaped, leading to a complex immune landscape whose ability to protect us from past and current strains is difficult to quantify^4,5^. Although much effort has been devoted to measuring individual antibodies and predicting the effectiveness of their combinations^6–9^, the inverse problem using a mixture’s collective behavior to characterize the antibodies within is intractable without a framework to enumerate all antibody–virus interactions. In this Article we create such a framework, which provides a unique perspective to computationally dissect mixtures.

To that end, we characterize antibody–virus interactions based on the techniques of antigenic cartography^10,11^ and antibody fingerprinting^12^. Antigenic cartography creates a low-dimensional map from hemagglutination inhibition titers that quantifies the tradeoffs in how potently and broadly sera can inhibit different groups of viruses. Although this technique imposes a structure for how sera can behave, it is unable to characterize the antibodies within a given serum nor predict the level of inhibition offered when sera are pooled together. Moreover, hemagglutination inhibition only characterizes antibodies binding to the head of influenza hemagglutinin (HA) and neglects antibodies targeting the HA stem, which generally inhibit a broader set of viruses^1^ and which are being assessed in clinical trials as therapeutics^13^.

A complementary method that partially offsets these drawbacks is antibody fingerprinting, which links the behavior of individual antibodies and antibody mixtures. The neutralization of large panels of antibodies is first clustered to identify patterns or ‘fingerprints’^12^. By applying this process in reverse, neutralization from polyclonal sera can be decomposed to identify constituent antibodies from the original panel.

In this Article we create a neutralization landscape that characterizes the interaction between HA-stem-targeting antibodies and influenza viruses. This approach pushes beyond cartography and fingerprinting in three key ways. First, as in cartography, we apply multidimensional scaling to antibody–virus measurements and project them into two dimensions (2D), but we do so at the level of individual antibodies rather than sera. By shifting the focus to antibodies, we quantify neutralization in absolute units without normalization factors, and we avoid the tendency of polyclonal sera to disrupt the map structure by drawing together antigenically distinct viruses. Moreover, whereas the composition of sera is generally unknown, and hence antigenic maps represent an amalgam of antibodies targeting multiple antigenic sites, the composition and binding site of an individual antibody can be precisely quantified, resulting in a more accurate and interpretable landscape.

Second, this landscape serves as a discovery space for new antibodies and viruses. We empirically demonstrate that a 2D distance function (or metric) characterizes the >1,000 antibody–virus interactions we measured. By positing that other stem antibodies and viruses will be well characterized on the observed landscape, we can enumerate the range of antibody behaviors. For example, we can visualize the potency–breadth tradeoff (quantifying how antibodies inhibiting more diverse viruses must have decreased neutralization) and predict the maximal potency of an antibody against any set of mapped viruses.

Finally, inspired by antibody fingerprinting, we develop a technique to decompose the collective neutralization from a mixture and characterize the antibodies within. Whereas previous efforts only detected specific patterns from known antibodies^14^, our approach considers a range of stem antibody behaviors extrapolated via the landscape. Using this rich set of behaviors, we determine the minimal number of stem antibodies (along with their full neutralization profile and stoichiometry) that could generate the observed signal from a mixture. Moreover, the neutralization landscape can remove the effects of non-HA-stem antibodies, and we validate such decompositions against 14 mixtures of HA head + stem antibodies. In this way, the neutralization landscape can peer into the influenza antibody response and quantify the stem antibodies within.

## Results

### Quantifying the spectrum of influenza antibody neutralization

Antigenic cartography utilizes metric multidimensional scaling to coalesce individual interactions (the ability of one antibody to inhibit one virus strain) into a global map^15^. As a helpful geographic analogy, multidimensional scaling transforms pairwise distances between cities to create a state map (Supplementary Fig. 1). When cities are replaced by viruses and antibodies, the same procedure generates a map where the concentration of an antibody required to neutralize a virus is solely dictated by its distance to that virus, with smaller distances signifying more potent neutralization.

**Fig. 1:**
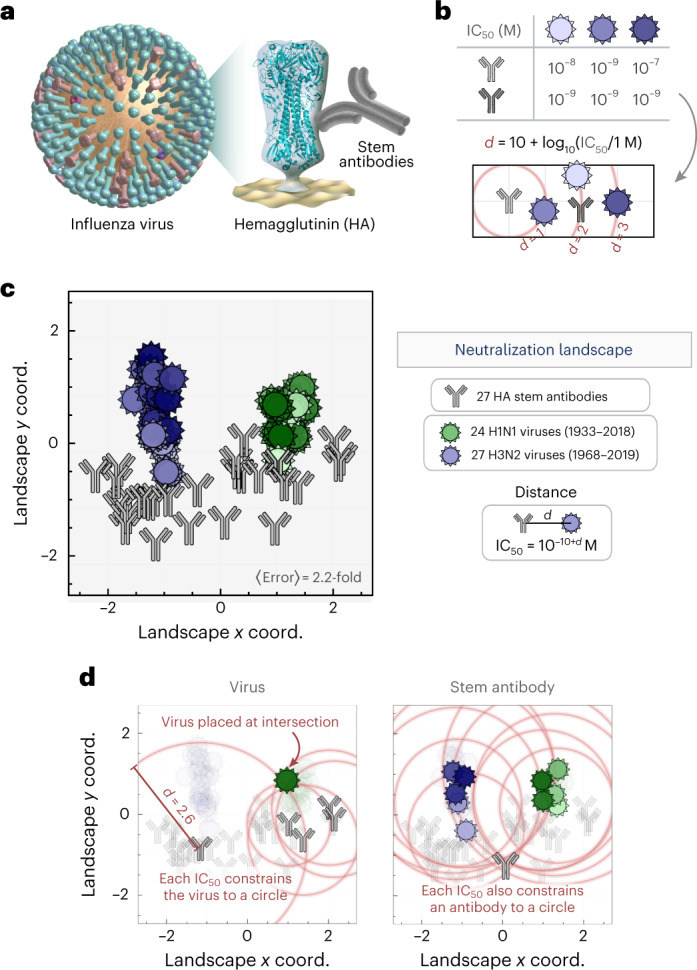
Neutralization landscape for the influenza hemagglutinin stem. **a**, Virus neutralization was measured for antibodies targeting the influenza HA stem. **b**, Example showing how neutralization is transformed into a distance *d* on the landscape. An antibody with greater neutralization (smaller IC_50_) against a virus is placed closer to that virus. The light-gray antibody is positioned 1, 2 or 3 units away from the viruses, and the dark-gray antibody is 1 unit away from all three viruses. **c**, Neutralization landscape of the HA stem quantifying the interactions between monoclonal antibodies (gray) and viruses (hues of green/blue, with darker hues representing more recent viruses). Throughout this work, landscapes are portrayed using a 2D Euclidean coordinate system where distance *d* between each antibody–virus pair corresponds to a neutralization IC_50_ = 10^−10 + *d*^ M, so that gridlines represent a 10× drop in neutralization. Average error represents the mean fold-difference between the landscape IC_50_ values and measurements. Supplementary Fig. 1 presents the raw data and the correspondence between shading and virus name; this color scheme is used consistently throughout this work. **d**, Examples of how a virus or antibody is positioned. Red circles represent the expected distance *d* = 10 + log_10_(IC_50_/1 M), and an antibody or virus must lie as close as possible to the intersection of all circles. Source data

We assembled a virus panel comprising 24 H1N1 influenza strains collected between 1933 and 2018 and 27 H3N2 strains collected from 1968 to 2019 (Supplementary Fig. 2 and Supplementary Table 1). Neutralization was measured against 27 HA-stem-targeting antibodies (Fig. 1a; 17 previously published in ref. 16 and 10 newly measured antibodies) representing major lineages of broadly neutralizing antibodies elicited by vaccination^17–19^. We determined the concentration of each antibody needed to neutralize every virus by 50% (the half-maximal inhibitory concentration, IC_50_), and the available reagents were sufficient to measure most antibody–virus pairs (1,148/1,377 = 85%, raw data are presented in Supplementary Fig. 1c). These measurements allow us to construct a neutralization landscape where the relative distance between an antibody and virus dictates the antibody’s potency (Fig. 1b). As more viruses and antibodies are added, they lock into a fixed configuration, aside from global translations, rotations and reflections.

Using these antibody–virus interactions, we created a neutralization landscape for the HA stem, with H1N1 and H3N2 viruses colored from lightest-to-darkest hues (oldest to more recent strains, Fig. 1c). A distance *d* between an antibody and virus translates into an IC_50_ of 10^−10 + *d*^ M (where 1 μg ml^−1^ = 6.6 × 10^−9^ M for the immunoglobulin-G (IgG) antibodies considered here), so that greater distance represents exponentially decreasing inhibitory action. We quantified the error of the antibody and virus coordinates by computing the fold error between the landscape IC_50_ values and the measured IC_50_ values for all antibody–virus pairs (for example, ${{{\mathrm{IC}}}}_{50}^{{{{\mathrm{Predict}}}}} = {10}^{ - 9}\,{{{\mathrm{M}}}}$ and ${{{\mathrm{IC}}}}_{50}^{{{{\mathrm{Measure}}}}} = {2} \times {10}^{ - 9}\,{{{\mathrm{M}}}}$ has a fold error of two), with a lower limit of 1-fold error for a landscape that perfectly represents the data (Methods). The 2D stem landscape had an 〈error〉 of 2.4-fold, comparable to the approximately 2-fold accuracy of the neutralization assay.

Surprisingly, when we remade the landscape in different dimensions, the error only decreased by 10% in 3D, although it more than tripled in 1D (Supplementary Fig. 3). Moreover, as described in the following section, the 2D landscape has better predictive power than higher dimensions. Accordingly, we opted to represent the data in 2D. We further showed that our method outperforms other dimensionality reduction techniques (Methods).

The resulting 2D landscape is described by 2 × (27 antibodies + 51 viruses) = 156 coordinates representing the 1,148 antibody–virus measurements (compressing the data to 156/1,148 = 15%). To visualize the structure of the data that enables this compression, we draw circles of radius *d* = 10 + log_10_(IC_50_/1 M) around several antibodies measured against a virus. This virus must lie as close as possible to all circles, and its location can be determined via least-squares minimization (Fig. 1d, left panel). Antibodies and viruses are treated symmetrically, so an antibody is similarly fixed using its neutralization against multiple viruses (Fig. 1d, right panel). We note that the circles shown in Fig. 1d represent a small fraction of available data, with each virus constrained by 20 measurements and each antibody constrained by 40 measurements, on average. Error analysis shows that antibody and virus coordinates are tightly determined (Supplementary Fig. 2 and Supplementary Table 1).

### Predicting the neutralization of new antibodies or viruses

The success of multidimensional scaling suggests that the interactions between stem antibodies and influenza viruses have a simple underlying structure. As with all dimensionality reduction techniques, this approach is expected to interpolate accurately (for example, given an antibody’s neutralization against several mapped viruses, we can predict its behavior against all other mapped viruses) yet extrapolate poorly (for example, predicting neutralization against a new antibody or virus). Previous efforts have focused exclusively on interpolation^10,20^, but we postulated that a neutralization landscape constrains the space of antibody–virus interactions sufficiently to make extrapolation possible.

**Fig. 2:**
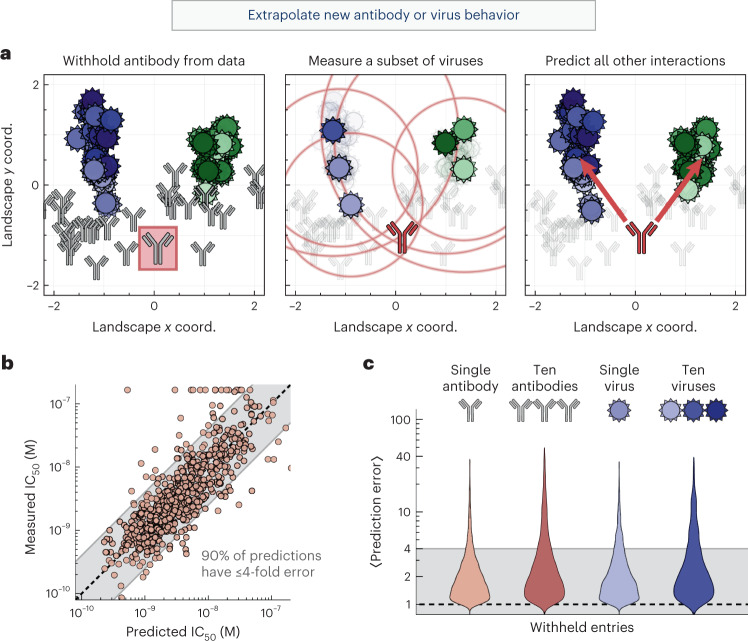
Extrapolating new antibody behavior. **a**, Left: we withhold an antibody (315-23-1C09, boxed in red) from our dataset and recreate the neutralization landscape. Middle: the location of the withheld antibody is triangulated on the new landscape using a few measurements. Right: the neutralization against the remaining viruses is predicted using antibody–virus distance. Gridlines represent a 10× drop in neutralization. **b**, The 600 predicted versus measured IC_50_ values after withholding every antibody in our panel. **c**, We withhold either antibodies or viruses (1 or 10 of each), triangulate each entity using a subset of measurements, and predict the remaining measurements. In **b** and **c**, the shaded band represents ≤4-fold error, where 1-fold error represents an exact prediction. Source data

To test this hypothesis, we withheld all measurements from one antibody and recreated the neutralization landscape. We then quantified whether some point on the map could still describe this antibody. In other words, do the unoccupied regions of the landscape predict the behavior of potential antibodies? Using six measurements, we triangulated the withheld antibody and compared its predicted versus measured neutralization against the remaining 45 viruses (Fig. 2a and Methods).

We repeated this analysis, withholding each of the 27 antibodies in turn and predicting the left-out antibody’s complete neutralization profile (taking special care with the bounded measurements; Methods). Collectively, 65% of the 600 predicted IC_50_ values had ≤2-fold error, and 90% had ≤4-fold error (Fig. 2b and left distribution in Fig. 2c). When we similarly withheld and triangulated a virus, we found that 80% of the resulting predictions had ≤4-fold error (Fig. 2c). Moreover, we found that a 2D landscape predicts a withheld antibody or virus better than lower or higher dimensions (Supplementary Fig. 3a).

As a more ambitious test, we next withheld multiple antibodies and viruses. We removed the ten most recent viruses (isolated between 2010 and 2020), representing the practical scenario where past strains are used to infer the behavior of future variants. In addition, we assessed whether the landscape remained stable when antibodies from an entire region were depleted. Thus, we removed ten antibodies (37% of our set) from either the left half or right half of the map. In each scenario, we triangulated every entry as described above, using six measurements to predict the remaining data. In all cases, we found that 80% of predictions had ≤4-fold error, demonstrating that the map can robustly infer new antibody or virus behavior (Fig. 2c).

In our initial dataset of 1,148 antibody–virus measurements, most predictions fell within 4-fold of the measurements (much smaller than the 2,000-fold range of IC_50_ values across the dataset). However, 16 measurements exhibited >10-fold error, suggesting that those few measurements may be outliers. To test this, we remeasured these 16 interactions and found that, upon remeasurement, their error decreased from 22-fold to 6-fold on average, much closer to the landscape predictions (Supplementary Fig. 4; all figures in this Article utilize these remeasured values). To test for false negatives, we also remeasured 54 interactions with <10-fold error (already in line with our predictions) and found that the measurements were mostly identical and their error minimally changed from 2-fold to 3-fold on average (Supplementary Fig. 4). Thus, by quantitatively analyzing the dataset with our landscape, we could identify and correct outliers.

Although the above analysis suggests that our specific dataset is well described by a 2D landscape, we cannot know how these results will generalize as more antibodies and viruses are added. In particular, stem antibodies that are not broadly neutralizing or viruses from other subtypes may require a landscape with different dimensionality or a different distance metric. As a first step to testing the generality of our approach, we analyzed an external dataset, where the neutralization of four additional stem antibodies not in our panel were measured against 13 viruses^21^. We first showed that triangulation with six viruses could predict the remaining measurements with 2.6-fold accuracy, demonstrating that these antibodies conform to the underlying structure of our landscape (Supplementary Fig. 5). We then used the positions of our mapped viruses to extend their dataset, predicting 36 new IC_50_ values for each antibody (Supplementary Fig. 5).

### Antibody–virus distance quantifies the potency–breadth tradeoff

Although it is well known that stem antibodies tend to neutralize a broader set of viruses than head antibodies^22^, precisely quantifying the inherent tradeoff between antibody potency and breadth remains an open problem. Using the neutralization landscape, we transform this challenging biological question into a straightforward geometry problem (Supplementary Fig. 1e).

A key mathematical property of the landscape is that the antibody–virus distance forms a metric. In other words, our intuition for Euclidean geometry applies—for example, the antibody with the most potent neutralization (lowest IC_50_) against two viruses would lie exactly between them, minimizing the distance to either virus.

This set-up is readily generalized to multiple viruses to answer a question that is intractable without a reference set for antibody behavior: how potently could any antibody neutralize *N* viruses? (Formally, what is the minimum ${{{\mathrm{IC}}}}_{50}^{{{{\mathrm{min}}}}}$ such that an antibody only exhibits ${{{\mathrm{IC}}}}_{50}{{{\mathrm{s}}}} \le {{{\mathrm{IC}}}}_{50}^{{{{\mathrm{min}}}}}$ against all *N* viruses?) On the landscape, this optimal antibody lies at the center of the smallest circle bounding all *N* viruses.

**Fig. 3:**
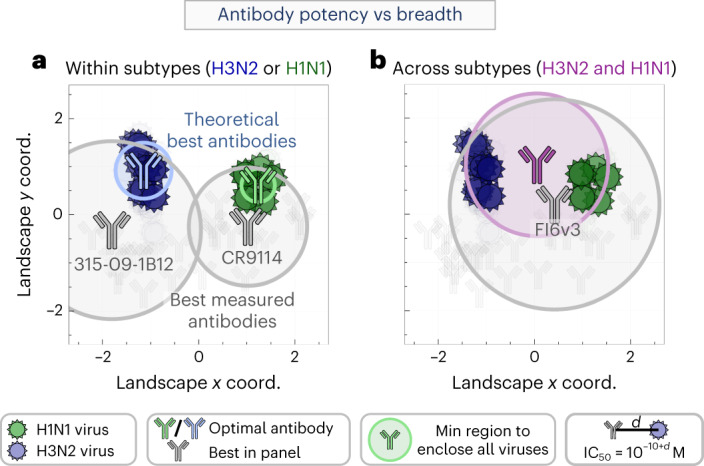
The neutralization profile of an optimal antibody against any set of mapped viruses. **a**, Positions of the hypothetical highest-potency antibodies with the smallest IC_50_ against all H1N1 (green) or H3N2 (blue) vaccine strains from the 2004–2005 to 2018–2019 seasons, compared to the best antibody in the panel (gray, labeled below with their names). A circle corresponding to the minimal IC_50_ enclosing all relevant viruses is drawn around each antibody. **b**, Predicting the highest-potency antibody (purple) that neutralizes both the H1N1 and H3N2 strains compared to the best antibody in our panel (gray). Source data

To demonstrate this process, we considered the optimal antibody targeting all H1N1 or H3N2 vaccine strains from the 2004–2005 to 2018–2019 seasons (Methods). The optimal H3N2-specific antibody (blue in Fig. 3a) has a predicted ${{{\mathrm{IC}}}}_{50}^{{{{\mathrm{min}}}}} = {4}\times {10}^{ - 10}\,{{{\mathrm{M}}}}$, around 20× better than the best antibody in our panel (gray antibody 315-09-1B12) with a measured ${{{\mathrm{IC}}}}_{50}^{{{{\mathrm{panel}}}}} = {70}\times {10}^{ - 10}\,{{{\mathrm{M}}}}$ (Fig. 3a). In contrast, the optimal H1N1-specific antibody (green) has ${{{\mathrm{IC}}}}_{50}^{{{{\mathrm{min}}}}} = {3}\times {10}^{ - 10}\,{{{\mathrm{M}}}}$, only 2.5× better than the ${{{\mathrm{IC}}}}_{50}^{{{{\mathrm{panel}}}}} = {10}\times {10}^{ - 10}\,{{{\mathrm{M}}}}$ from our best antibody (gray, antibody CR9114). If each point on the map describes a potential antibody, then groups searching for better stem antibodies against these viruses should expect only marginal gains from additional H1N1-targeting antibodies, but far greater potential for finding more potent H3N2-targeting antibodies.

We can similarly assess how well a single antibody simultaneously neutralizes both the H1N1 and H3N2 vaccine strains. Because of the differences between these two subtypes, we expect that this enlarged breadth must come at the cost of decreased potency. Indeed, the neutralization landscape predicts that a stem antibody can only exhibit ${{{\mathrm{IC}}}}_{50}^{{{{\mathrm{min}}}}} = {30}\times {10}^{ - 10}\,{{{\mathrm{M}}}}$ against these vaccine strains, at least 10× less potent than the ${{{\mathrm{IC}}}}_{50}^{{{{\mathrm{min}}}}}$ values for the H1N1-specific or H3N2-specific antibodies described above. As expected, the best panel antibody that simultaneously neutralizes these H1N1 and H3N2 viruses has ${{{\mathrm{IC}}}}_{50}^{{{{\mathrm{panel}}}}} = {140}\times {10}^{ - 10}\,{{{\mathrm{M}}}}$ (FI6v3); that is, ~5× worse that the predicted optimal antibody (Fig. 3b). These results emphasize that even broadly neutralizing antibodies may have a ‘neutralization ceiling’ against sufficiently diverse viruses, and that there is an inherent cost to neutralizing more strains.

This process is readily extended to any group of viruses, as well as to the more general question of how potently *N*_1_ antibodies could neutralize *N*_2_ viruses. Using the landscape, we can not only compute the optimal IC_50_, but also the specific neutralization profile against each virus on the panel.

As a technical note, a metric requires a triangle inequality. Because we only define the distance *d*_Ab–V_ = 10 + log_10_(IC_50_/1 M) between an antibody (Ab) and virus (V), the usual triangle inequality becomes the quadrilateral inequality

1 $${d}_{{{{\mathrm{Ab}}}} - {{{\mathrm{V}}}}} \le {d}_{\overline {{{{\mathrm{Ab}}}}} - {{{\mathrm{V}}}}} + {d}_{\overline {{{{\mathrm{Ab}}}}} - {\overline{\mathrm{V}}}} + {d}_{{{{\mathrm{Ab}}}} - {\overline{\mathrm{V}}}}$$

where ${\overline{\mathrm{Ab}}}$ and ${\overline{\mathrm{V}}}$ represent any other antibody or virus (Supplementary Fig. 6a). As with the traditional triangle inequality, this relationship codifies the notion that the distance between any Ab and V must be shorter than the next shortest route through ${\overline {{{{\mathrm{Ab}}}}}}$ and ${\overline{\mathrm{V}}}$. Altogether, there are 400,000 combinations of Ab, ${\overline {{{{\mathrm{Ab}}}}}}$, V and ${\overline{\mathrm{V}}}$ that can be directly tested against equation (1) using the antibody–virus measurements (without requiring the neutralization landscape). We find that this inequality is satisfied in 99% of cases, demonstrating that our measurements are well described by the Euclidean metric. This empirical observation must be continually affirmed with future measurements.

### Isolating the neutralization of a stem antibody within a mixture

**Fig. 4:**
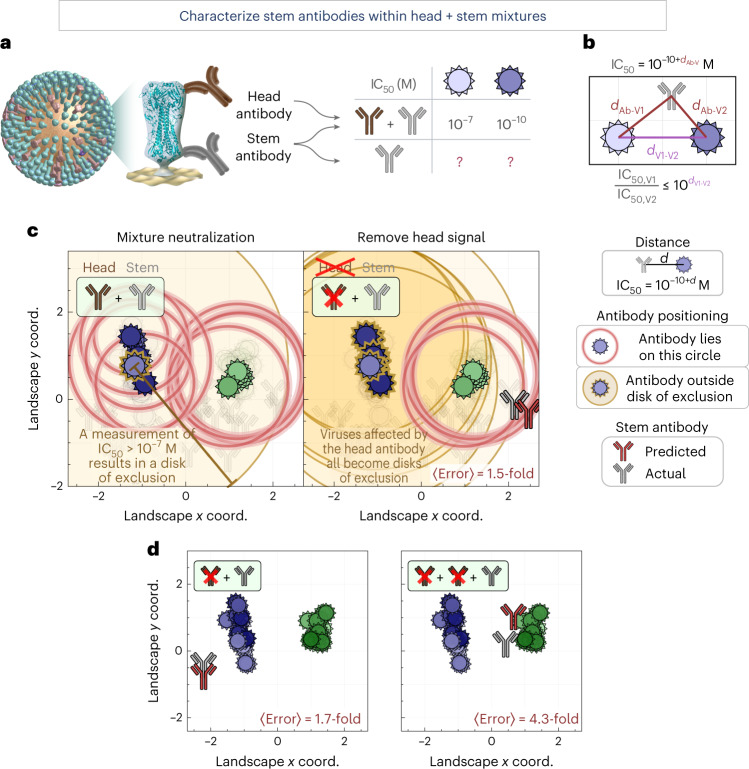
Characterizing a single stem antibody within head + stem mixtures. **a**, We created mixtures of two or three antibodies targeting the HA head and stem. Using a mixture’s neutralization titers, we predict the behavior of the stem antibody within. **b**, The virus–virus distance on the landscape constrains how differently any stem antibody can neutralize two viruses. **c**, Left: the neutralization from a head + stem mixture (#7 in Supplementary Fig. 10) cannot be described by any point on the landscape due to the head antibody. The gold disk represents a lower bound on neutralization (for example, IC_50_ > 10^−7^ M, *d* > 3); an antibody should lie outside all gold disks but on every red circle (representing exact IC_50_ values). Right: after using equation (2) to remove the neutralization from the head antibody, the IC_50_ values of all blue viruses become lower bounds (gold disks), and we can infer the position of the stem antibody (red). The insets at the top left show the number of head (brown) and stem (gray) antibodies in each mixture, and whether the head signal has been removed (antibody icon crossed out). **d**, Examples showing additional mixtures combining one stem antibody with one or two head antibodies (additional decompositions are shown in Supplementary Fig. 10). Average error quantifies the fold-difference between the predicted antibody’s IC_50_ values and measurements. Source data

By enumerating the behaviors of a stem antibody, the neutralization landscape can also detect neutralization patterns from non-stem antibodies and remove those signals. For example, given the combined neutralization from an HA head + stem antibody mixture, we can isolate the stem-derived neutralization (Fig. 4a). This represents a key step towards serum deconvolution (characterizing the epitopes and neutralization profiles of antibodies within serum), which is a necessary step in the many ongoing efforts to elicit broad antibodies that can grant potent and durable protection against infection.

Although antibody–virus distance on the landscape corresponds to neutralization, the distance between two viruses (V1 and V2) can be interpreted as constraining how differently any stem antibody can neutralize both strains. In the extreme example where these viruses have identical HA stems and lie on the same coordinate (*d*_V1–V2_ = 0), every stem antibody will identically neutralize V1 and V2. Thus, if a head + stem antibody mixture neutralizes V1 far more potently than V2 (for example, with IC_50_ values of 10^−10^ M and 10^−7^ M, respectively), this discrepancy must be caused by the head antibody increasing the mixture’s neutralization (decreasing its IC_50_) against V1. The stem antibody alone should exhibit the same IC_50_ value of ≥10^−7^ M against both viruses (a value larger than 10^−7^ M is possible if the head antibody increases the mixture’s neutralization against both viruses).

More generally, given a distance *d*_V1–V2_ between two viruses, any stem antibody (Ab) will obey |*d*_Ab–V1_ − *d*_Ab–V2_| ≤ *d*_V1–V2_ (Fig. 4b) or equivalently

2 $${\frac{{{{{\mathrm{IC}}}}_{50,\,{{{\mathrm{V}}}}1}}}{{{{{\mathrm{IC}}}}_{50,\,{{{\mathrm{V}}}}2}}} \le {10}^{d_{{{{\mathrm{V}}}}{1}-{{{\mathrm{V}}}}2}}}.$$

Given the combined neutralization from a head + stem mixture, we consider every pair of measurements (IC_50, V1_, IC_50, V2_) and determine whether their ratio exceeds ${10}^{d_{{{{\mathrm{V}}}}{1} - {{{\mathrm{V}}}}2}}$. If equation (2) is satisfied, the measurements remain unchanged. Otherwise, the smaller value (say IC_50, V2_) is bounded below as per equation (2) (IC_50, V2_ ≥ IC_50,V1_ × ${10}^{ - d_{{{{\mathrm{V}}}}1-{{{\mathrm{V}}}}2}}$, Supplementary Fig. 6b,c; see Methods for how we account for noise in the measurements by requiring more than a 10-fold discrepancy).

To test this process, we created nine antibody mixtures (seven containing 1 head + 1 stem antibodies and two containing 2 head + 1 stem antibodies) and measured them against our virus panel (*N* = 321 data points, Supplementary Fig. 7). As an example, we consider one mixture where the stem antibody is H1N1-specific and does not neutralize any H3N2 viruses, whereas the head antibody neutralizes a few H1N1 and H3N2 strains (head antibody C05 + stem antibody CR6261). In combination, these two antibodies moderately neutralized some H3N2 viruses with an IC_50_ of ≤10^−8^ M (Fig. 4c, left panel, red circles around the blue viruses), whereas others showed no detectable neutralization (a gold disk around a virus represents the lower bound measurement, IC_50_ > 10^−7^ M, outside our range of detection). The stem antibody should lie outside any gold disk while lying on the red circles representing IC_50_ values within our range of detection. As expected, no point on the landscape can satisfy these constraints in the left panel of Fig. 4c, demonstrating that a stem antibody alone cannot give rise to the neutralization from this head + stem mixture.

As described above, we use the neutralization landscape to remove the effects of the head antibody. Given the proximity of the H3N2 virus with no detectable neutralization (gold disk) to the H3N2 viruses with moderate neutralization (red circles), we correctly predict that the head antibody is responsible for this moderate neutralization. Thus, we apply equation (2), which increases the H3N2 IC_50_ values and changes them to lower bounds (represented by gold disks in the right panel of Fig. 4c). Notably, the H1N1 IC_50_ values were unchanged by this process, because the stem antibody’s neutralization dominated against these viruses, and hence the mixture’s H1N1 neutralization obeyed the constraints of the landscape.

With the head neutralization removed, we can triangulate the stem antibody on our map, as discussed in the previous section (Fig. 2a). In this way, we can characterize a stem antibody without knowing its individual neutralization profile nor the number or properties of the head antibodies in the combination. For our example mixture, the predicted stem antibody (Fig. 4c, red antibody) lies near the true position of the stem antibody (gray), with an average 1.5-fold error between the predicted and measured IC_50_ values across the virus panel. We repeated this analysis for our nine antibody mixtures, combining either one or two head antibodies with a stem antibody, and found an 〈error〉 ranging between 1.5- and 5.2-fold (mean of 3.4-fold; Fig. 4d and Supplementary Fig. 10).

### Characterizing antibody mixtures with multiple stem antibodies

Given the range of behaviors for a single stem antibody (represented by each point on the landscape), we can predict how multiple stem antibodies act in concert, paving the way to explore a polyclonal antibody response (the purview of this section). In the reverse direction, we can use the collective neutralization from multiple stem antibodies to determine the number, stoichiometry and neutralization profiles of the constituent antibodies (discussed in the following section).

**Fig. 5:**
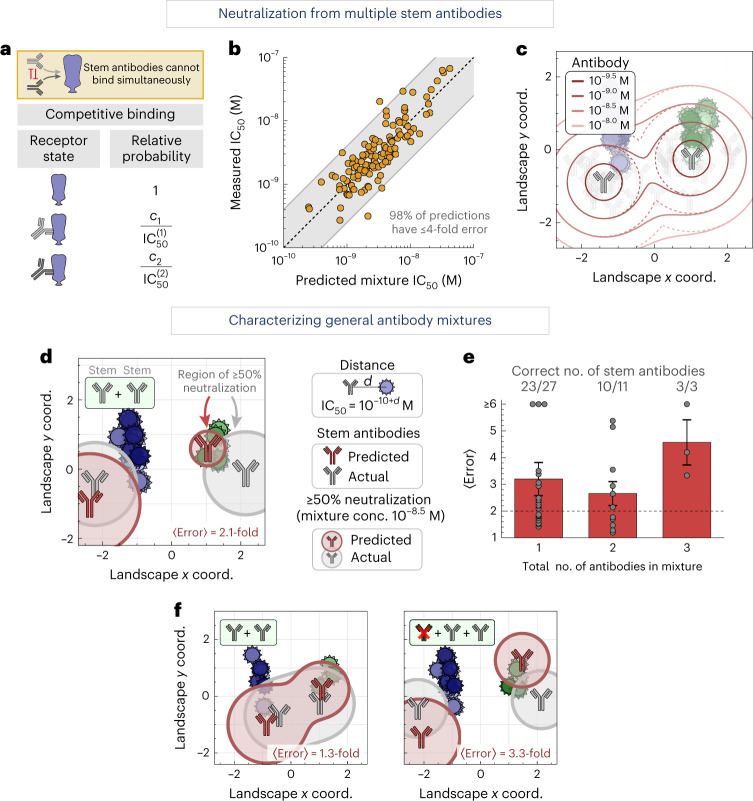
Characterizing mixtures with multiple stem antibodies. **a**, Biophysical model for mixtures of stem antibodies binding competitively to an HA monomer. **b**, Stem + stem mixtures were measured against the virus panel. Mixture IC_50_ values are predicted using the neutralization of each individual antibody. The gray shading represents ≤4-fold error, where 1-fold error represents an exact prediction. **c**, Regions of ≥50% neutralization for a two-antibody mixture (outlined in solid lines) versus the neutralization of each individual antibody (dashed lines). The legend shows the concentration of each antibody in the mixture. **d**, Using a mixture’s neutralization titers, we predict the number, stoichiometry and neutralization profiles of the stem antibodies within. One such stem + stem mixture is shown (gray antibodies, mixture 8 in Supplementary Fig. 10), together with the predicted decomposition (red). Circles around each antibody represent ≥50% virus neutralization when the total antibody concentration is 10^−8.5^ M, while factoring in antibody stoichiometry within the mixture (Methods). Average error represents the fold-difference between the collective neutralization predicted by the inferred stem antibodies and the measured neutralization of only the stem antibodies in the mixture, with 〈error〉 = 1 representing exact predictions. **e**, Mean + s.e.m. for the decomposition of 27 monoclonal antibodies, 11 mixtures containing two antibodies (stem + stem or head + stem) and three mixtures containing three antibodies (head + head + stem or head + stem + stem). The fractions of decompositions that predicted the correct number of stem antibodies are shown above each bar. Experimental noise is ~2-fold (dashed line). **f**, Examples showing additional mixtures combining two stem antibodies with and without a head antibody (additional decompositions are shown in Supplementary Fig. 10). Neutralization of two nearby antibodies is expanded because either can neutralize a virus, analogous to the solid lines in **c**. The insets at the top left show the number of head (brown) and stem (gray) antibodies in each mixture. Source data

To that end, we construct a biophysical model that calculates a mixture’s neutralization based on the neutralization of each individual antibody. Because the stem antibodies in our panel all target the same region of the HA stem^17,23,24^, we treat their binding as competitive, where only one antibody can bind to an HA monomer at a time (Fig. 5a). For two stem antibodies with individual neutralizations ${{{\mathrm{IC}}}}_{50}^{(1)}$ and ${{{\mathrm{IC}}}}_{50}^{(2)}$, a mixture containing a fraction *f*_1_ of the first antibody and *f*_2_ = 1 − *f*_1_ of the second antibody will have

3 $${{{\mathrm{IC}}}}_{50}^{{{{\mathrm{Mixture}}}}} = {\left( {\mathop {\sum}\nolimits_j {f_j/{{{\mathrm{IC}}}}_{50}^{(j)}} } \right)^{ - 1}},$$

with this same equation holding for mixtures containing more antibodies (Methods).

To test this competitive binding model, we created four stem + stem antibody mixtures and measured them against our virus panel (*N* = 165 data points). Using the individual IC_50_ values against each virus, we find tight agreement between the predicted and measured mixture IC_50_ values, with 98% of predictions exhibiting ≤4-fold error (Fig. 5b). On the landscape, such mixtures will neutralize a larger region (Fig. 5c, solid lines) than either antibody alone (dashed lines). In this way, we can use the competitive binding model to predict and visualize the behavior of general stem mixtures.

### Characterizing the stem antibodies within general mixtures

By combining the results from the two previous sections, namely removing the neutralization of head antibodies and enumerating the behavior of multiple stem antibodies, we can decompose antibody mixtures with multiple head or stem antibodies. In essence, decomposition detects neutralization signatures that are impossible for a monoclonal antibody to achieve (for example, potent neutralization against viruses far apart on the landscape). In such cases, we search for the minimum number of antibodies that give rise to the apparent neutralization profile.

As an example, we measured the collective neutralization from a mixture of two stem antibodies against our virus panel (Fig. 5d, gray antibodies; CR6261 + CR8020, 50/50% composition). The algorithm scans through all possible configurations of *n* = 1, 2, 3… antibodies on the landscape and determines which one best describes the mixture, terminating once the fold error no longer appreciably decreases with additional antibodies (Supplementary Figs. 8 and 9 and Methods). This correctly predicted two stem antibodies (Fig. 5d, red), although with a 10/90% composition. The circle surrounding each antibody represents this fractional composition, so that the gray circles have the same radius whereas the red circle of the antibody on the left of the landscape (representing 90% composition) is larger than the red circle of the antibody on the right (10% composition). The areas covered by these circles represent ≥50% neutralization when the total mixture concentration equals a fixed amount we chose as 10^−8.5^ M. The deviation between the predicted and actual coordinates is partially compensated by predicting an uneven composition, so that the average fold error between the measured and predicted IC_50_ values against all viruses is 1.9-fold, comparable to experimental error. This demonstrates that the antibody response can be partially degenerate—where different mixtures exhibit similar behavior—as both the 50/50% composition of gray antibodies and the 90/10% composition of red antibodies exhibit similar neutralization.

We performed a similar analysis for all our antibody mixtures, which included (1) 27 monoclonal stem antibodies, (2) 11 mixtures containing two antibodies (four stem + stem and seven head + stem) and (3) three mixtures containing three antibodies (two head + head + stem and one head + stem + stem; Supplementary Fig. 7). As above, we blinded ourselves to both the number and type of antibodies in each mixture, computationally removed any head antibody neutralization (even for mixtures containing only stem antibodies), determined which set of stem coordinates best characterized the neutralization profile, and compared the resulting neutralization against the true mixture compositions.

Across all mixtures, the collective neutralization from the stem antibodies was well characterized by these decompositions, with ~3-fold error for the monoclonal antibodies and two-antibody mixtures and 4‒5-fold error for the three-antibody mixtures (Fig. 5e,f). For 23/27 monoclonal antibodies, the decomposition correctly predicted a single stem antibody, although four antibodies were overfit as two-antibody mixtures due to deviations between landscape distances and measurements. Decompositions for 10/11 of the two-antibody mixtures and 3/3 of the three-antibody mixtures predicted the correct number of stem antibodies. Notably, in every mixture containing more than one stem antibody, we identified the unique antibodies, even when they were as close as 1.5 units apart on the landscape (mixtures 8–11 and mixture 14 in Supplementary Fig. 10).

For the 13/14 mixtures with the correct number of imputed stem antibodies, we can further assess the predicted stoichiometry and neutralization profile of each stem antibody within the mixture. Because we remove neutralization from HA head antibodies, our algorithm computes the abundance of each remaining stem antibody relative to the stem-directed response. Hence, the eight mixtures with a single stem antibody were (by definition) correctly predicted to have that antibody comprise 100% of the stem response (Supplementary Fig. 10d). The remaining mixtures all had two stem antibodies with 50/50% stem-targeting composition; four mixtures were predicted with ~75/25% and one with 90/10% stoichiometry. In addition, the distance between the predicted stem antibodies and their true landscape positions was 0.8 ± 0.4 map units (corresponding to six-fold deviations in IC_50_), which we note is relatively small compared to the 2,000-fold range of IC_50_ values across our dataset.

Collectively, these results suggest that, in most cases, a mixture’s neutralization profile can uniquely identify the number of stem antibodies within (36/41 ≈ 90% of cases). However, distinct mixtures can give rise to similar neutralization profiles, even when measured against a panel of 51 diverse viruses. The frequency of such degenerate mixtures, as well as the positions of additional viruses whose measurements would break these degeneracies, can be quantified by enumerating all possible mixtures on the landscape.

## Discussion

Since the advent of Fourier analysis in the 1800s, the ability to break signals into simple underlying components has revolutionized scientific disciplines ranging from complex analysis to image reconstruction. Recently, live cells were imaged using six fluorescent probes whose emission spectra heavily overlapped, so that the net luminescence was a cacophony of signals^25^. By characterizing each probe’s spectrum, the total signal could be unmixed, enabling six regions of live cells to be simultaneously imaged. In the context of antibody–virus interactions, the challenge of unmixing lies both in enumerating the spectrum of antibody–virus behaviors as well as in decomposing the collective inhibition of multiple antibodies.

The neutralization landscape we create quantifies the limits of antibody–virus interactions. Universal vaccine efforts aiming to elicit broadly neutralizing HA-stem antibodies should consider how broad they want this response to be, given the inverse relationship between breadth and potency. As another application, identifying which amino acids in the HA stem are associated with changes in virus antigenicity (that is, changes in the virus position in the neutralization landscape) will facilitate the design of HA-stem-based vaccines able to elicit specific immunological phenotypes.

By systematically enumerating the range of behaviors for individual stem antibodies, the landscape can decompose simple polyclonal mixtures—even if they include antibodies binding to other epitopes such as the HA head—and quantify their fractional composition and neutralization profiles. Fundamentally, the information driving these predictions is in the positions of the viruses on the landscape. When a mixture’s neutralization diverges from the possible profiles of a monoclonal antibody, it not only suggests that the mixture must be polyclonal, but also presents a way to quantify the functional properties of the antibodies within the mixture.

With this approach, we take a step towards one of the central challenges in immunology, namely, using the collective neutralization from a mixture of antibodies to characterize the specific antibodies within. Although in this work we only decomposed mixtures with two or three antibodies, the success of the similar antibody fingerprinting methodology^12^ suggests that our approach can be applied to more complex polyclonal sera. Indeed, recent studies have shown that the human antibody response against one strain of influenza is often dominated by ≤5 antibody clonotypes^26^ and in extreme cases by approximately one antibody^27^, and such ‘approximately monoclonal’ sera could be decomposed to characterize the dominant antibodies within. This opens a number of applications, including the following. (1) HA-stem vaccine performance could be quantified in terms of both the fraction of elicited antibodies that are on-target as well as the neutralization profile of those antibodies^28–32^. (2) Combining a neutralization landscape with a binding landscape (using antibody–virus dissociation constants) could quantify both neutralizing and non-neutralizing components of an antibody repertoire^33^. (3) Given the inherently limited supply of each serum sample, we could rationally design the closest approximating antibody mixture using known antibodies, enabling broader studies of promising mixtures and facilitating the development of therapeutics.

This decomposition is inherently limited by the diversity of viruses used to probe a mixture. Our approach uses each virus as a ‘sensor’ for nearby antibodies, so viruses should be widely spaced across the landscape to detect all possible antibodies. Due to experimental noise and the inaccuracy of the 2D representation, decomposition may only detect the dominant and distinct antibody signatures. An antibody comprising a small fraction of serum will minimally affect its neutralization and hence cannot be reliably detected. Moreover, antibodies with similar neutralization profiles may be represented by a single effective antibody. These cases add to degeneracy—a highly understudied feature of the antibody response—where combinations of different antibodies give rise to similar functional responses.

An open question is whether the 2D Euclidean landscape presented here will suffice as more viruses and antibodies are added to the map. More complex datasets may require higher-dimensional maps, a more complex metric or separate maps for antibodies binding to different antigenic sites.

Although the HA-stem neutralizing antibodies used in this study all bind to the canonical stem super-epitope^17,23,24^, antibodies targeting a new membrane-proximal stem epitope have recently been discovered^34,35^. Future work can explore whether their behavior is captured by the existing landscape, or if a separate map is required for each epitope. It also remains to be seen whether there are portions of the landscape that antibodies or viruses cannot occupy (for example, because of viral fitness or antibody polyreactivity).

Looking forward, the analysis presented here serves as a stepping stone to track the stem antibody response over time and predict how antibody repertoires respond to a pathogen. How will the stem response evolve after multiple vaccinations or infections, and is there a path dependence to antibody development or is all the relevant information contained within the current antibody repertoire^27,36–40^? The neutralization landscape reframes this biological problem into a geometry problem, where antibody evolution can be studied as a dynamical system with perturbations imposed by vaccinations and infections.

## Methods

The following sections briefly describe the steps to create the neutralization landscape and decompose antibody mixtures. The Supplementary Information contains more extensive descriptions.

### Measuring virus neutralization

We utilized existing antibody–virus neutralization measurements from ref. 16 (which measured (17 antibodies) × (49 viruses)) with new antibody–virus measurements carried out in this work. The development of replication-restricted reporter (R3) influenza viruses has been described in ref. 16. Briefly, the influenza PB1 viral segment was modified to encode a fluorescent reporter (mKate2 or TdKatushka2S), which replaced most of the coding region of the PB1 gene. R3 viruses can be propagated only in cells expressing PB1 *in* *trans*. For the influenza neutralization assay, PB1-expressing MDCK-SIAT1 cells suspended in OPTIMEM (Thermo Fisher) were seeded in 384-well plates (Greiner) at 150,000 cells ml^−1^ (20 μl per well), 1–2 h before infection. A 25-μl volume of each neutralization mixture, consisting of equal parts pre-titrated R3 influenza virus and four-fold serial dilutions of antibodies prepared in OPTIMEM containing 2 μg ml^−1^ TPCK-treated trypsin (Millipore Sigma), was transferred to wells in quadruplicate. The initial antibody concentrations after virus dilution were 25 μg ml^−1^.

Control wells of virus-alone and diluent-alone were included on each plate. Fluorescent foci were counted at 18–24 h post infection using a Celigo instrument (Nexcelom) with customized red filter to detect mKate2/TdKatushka2 reporter signals. Percent neutralization was calculated by constraining the virus-alone control to 0% and the diluent-alone control to 100% neutralization. We fit a curve of antibody concentration versus neutralization using a four-parameter nonlinear model in Prism (GraphPad), which determined the 50% inhibitory concentration, IC_50_. No statistical methods were used to pre-determine sample sizes. We included as many antibodies and viruses as possible, given the available reagents.

### Constructing the neutralization landscape

We transformed each neutralization IC_50_ into map distance (*d* = 10 + log_10_(IC_50_/1 M)) and performed multidimensional scaling, numerically minimizing the mean-squared difference between each antibody–virus pair’s landscape distance and desired distance. The resulting 2.2-fold error (shown in the bottom right of Fig. 1c) means that given any measured IC_50_ between an antibody–virus pair, the corresponding neutralization determined by the landscape will lie between IC_50_/2.2 and IC_50_ × 2.2, on average.

### Decomposing defined antibody mixtures

Using the neutralization landscape, we can decompose the collective neutralization from antibody mixtures to characterize the individual antibodies within. We first use the landscape to remove the neutralization signature of antibodies that do not target the HA stem. Next, we determine the optimal antibody mixtures (combination of points on the map) that recapitulate the resulting neutralization profile. Decomposition proceeds by characterizing a mixture using an increasing number of antibodies, halting once the addition of another antibody no longer markedly decreases the decomposition error.

### Reporting summary

Further information on research design is available in the Nature Portfolio Reporting Summary linked to this Article.

### Supplementary information

Supplementary Information Supplementary Discussion, Figs. 1–10 and Table 1.

Reporting Summary

Supplementary Data 1 Source Data for Supplementary figures.

### Source data

Source Data Fig. 1 Statistical source data.

Source Data Fig. 2 Statistical source data.

Source Data Fig. 3 Statistical source data.

Source Data Fig. 4 Statistical source data.

Source Data Fig. 5 Statistical source data.

## Data Availability

Details on how the experimental data were obtained are provided in the Methods. The final antibody–virus neutralization dataset used in this work is provided in the source data as well as in the associated GitHub repository (https://github.com/TalEinav/NeutralizationLandscape)^41^. Source data are provided with this paper.

## Code availability

The associated GitHub repository (https://github.com/TalEinav/NeutralizationLandscape) contains a Mathematica notebook that recreates all plots and analysis in this work^41^.
