# Supplementary material for: Harnessing low dimensionality to visualize the antibody–virus landscape for influenza
Source: Nat Comput Sci. 2022 Dec 30;3(2):164–73. doi: 10.1038/s43588-022-00375-1 (PMC10766546; doi:10.1038/s43588-022-00375-1)
Supplement: Supplementary file 1 — Supplementary Discussion, Figs. 1–10 and Table 1. [file 43588_2022_375_MOESM1_ESM.pdf]

nature computational science

Article

https://doi.org/10.1038/s43588-022-00375-1

Harnessing low dimensionality to visualize the antibody–virus landscape for influenza

In the format provided by the authors and unedited

A. Constructing the Neutralization Landscape **2**
Applying Multidimensional Scaling to Create the Neutralization Landscape 2
Applications and Limitations of the Neutralization Landscape 4
Computing the Error of a Neutralization Landscape 6
Utilizing Monoclonal Antibodies to Create the HA Stem Neutralization Landscape 6
Comparison with other Dimensionality Reduction Methods 7
Visualizing Virus Evolution using Landscape Positions 8
Dimensionality of the Landscape 12
Extrapolating the Behavior of New Antibodies 14
Flagging Potential Outliers in a Dataset 14
Adding New Antibodies or Viruses to the Map 15
The Triangle Inequality, Antibody-Virus Distance as a Metric 16
Removing Head Antibody Neutralization 18
The Tradeoff between Antibody Potency and Breadth 20

B. Modeling Combinations of Antibodies **21**
Overview of Antibody Mixtures 21
Collective Neutralization of Stem Antibody Mixtures is Well Characterized by Competitive Binding 21

C. Decomposing Defined Antibody Mixtures **23**
Why Polyclonal Mixtures need to be Represented using Multiple Points 23
Defining Decomposition Error 24
Choosing the Subset of Viruses for Decomposition 24
Overview of the Decomposition Algorithm 25
Results from Decomposing Antibody Mixtures 27

The GitHub repository (https://github.com/TalEinav/NeutralizationLandscape) associated with this manuscript also includes:

- *(1) Neutralization Landscape Coordinates for the HA Stem.csv*
- *(2) Neutralization Data for Monoclonal Antibodies and Mixtures.csv*
- *(3) Decomposition Results.csv*
- *(4) Virus HA Sequence Alignment.txt*
- *(5) A Mathematica notebook that recreates the Neutralization Landscape and all analysis*

A. Constructing the Neutralization Landscape

Applying Multidimensional Scaling to Create the Neutralization Landscape

To understand the utility of multidimensional scaling (MDS), we first introduce a useful analogy using a more familiar geographic map. Figure S1A shows a map of Texas along with several of its major cities. Using the scale bar at the top-right of this figure, it is straightforward to create a table of the distances between every pair of cities, as shown at the bottom of the panel.

Now consider the inverse scenario: given the distances between every pair of cities, how can you create a map of Texas? This is precisely the problem that multidimensional scaling solves. A simple algorithm is to choose random starting locations for each city and use numerical minimization techniques to minimize an error function such as $\sum_{j,k\in\text{cities}}(d_{jk}^{\text{Map}}-d_{jk}^{\text{Actual}})^2$ until the distance d_{jk}^{Map} between cities j and k on the map matches their actual distance d_{jk}^{Actual}. This problem is also known as the sensor network localization problem, and it has been well studied in terms of how many distance measurements are required and whether the result is robust to noise (*1*).

Metric multidimensional scaling presumes there is an underlying structure for these distances. This is clear in our geographic analogy; for example, if a new city was drawn on the map, it could not simultaneously lie 10 km from Austin and 10 km from Houston, since those two cities are 150 km apart. The key insight from Smith *et al.* was that antibody-virus interactions may possess a similar underlying structure that could be exploited to create an analogous map (*2*). The ability of an antibody to inhibit a virus would be inversely proportional to its distance from that virus, with a smaller distance implying a more potent antibody. In this context, each row in the table represents an antibody, each column a virus, and each entry measures the inhibition of this antibody against the virus through a metric such as binding affinity, HAI, or neutralization.

Figure S1B shows an example that can be exactly mapped in 2D. The Euclidean distance d between every antibody and virus (measured from the centers of their respective icons) corresponds to the 50% inhibitory concentration IC_{50} (at which half of the virus is neutralized) of 10^{-10+d} Molar. A distance of $d=0$ between an antibody and virus represents an $IC_{50}\leq10^{-10}$ M [which technically makes antibody-distance a pseudometric, since $d=0$ is not uniquely defined]. Since antibodies rarely bind with a dissociation constant less than 10^{-10} M (*3*, *4*), and because neutralization cannot occur without binding, there is a minimal loss in resolution from imposing this lower bound (which can be decreased further if needed). An antibody at a distance of $d=1$ from a virus exhibits an $IC_{50}=10^{-9}$ M, an antibody with distance $d=2$ exhibits an $IC_{50}=10^{-8}$ M, and so on, with a larger IC_{50} indicating a less potent antibody.

Multidimensional scaling proceeds by minimizing the average error between the predicted and measured IC_{50} values, $1/N \sum_{j\in\text{antibodies},k\in\text{viruses}}(d_{jk}^{\text{Predicted}}-d_{jk}^{\text{Measured}})^2$, where N equals the number of measurements, $d_{jk}^{\text{Predicted}}$ represents the predicted map distance between antibody j and virus k, and $d_{jk}^{\text{Measured}}=10+\log_{10}(IC_{50}^{(j,k)})$ denotes the experimentally measured IC_{50} converted into map distance

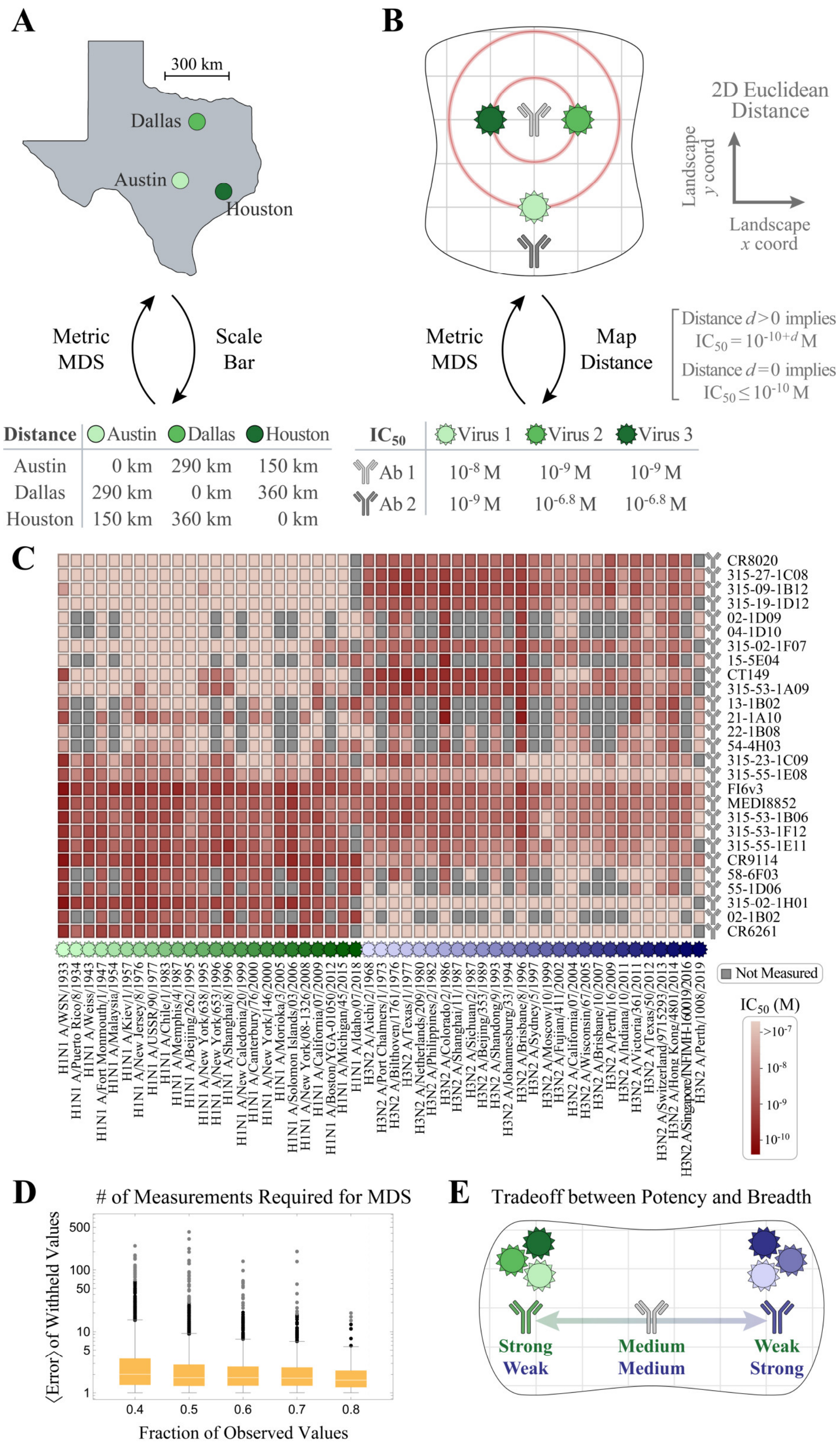

Supplementary Figure 1. Mapping antibody-virus interactions via multidimensional scaling. (A) Metric multidimensional scaling (MDS) used in a geographic example to determine a map of Texas from a table of distances between cities. (B) In the context of antibody-virus interactions, each row of this table represents an antibody while each column represents a virus, with the entry of the table denoting the antibody's neutralization against the virus. Metric MDS determines coordinates for the antibodies and viruses, where a 2D Euclidean distance of 0 represents an

$IC_{50} \leq 10^{-10}$ M while positive distance d represents an $IC_{50}=10^{-10+d}$ M between any antibody-virus pair. Thus, the top antibody would neutralize all viruses within the smaller circle of radius 1 by at least 50% when at a concentration of 10^{-9} M, and it would neutralize all viruses within the larger circle of radius 2 by at least 50% when present at a concentration of 10^{-8} M. (C) Dataset of 27 stem antibodies and 51 viruses used to create the Neutralization Landscape in Figure 1C. (D) We withhold values from the antibody-virus data matrix, perform multidimensional scaling using a fraction of observed valued (x-axis), and compare the fold-error of the withheld values to the landscape predictions (y-axis). Distributions show results from five runs (from left-to-right, these make n=2576, 1981, 1380, 781, and 187 predictions); each gold box spans from the 25%-75% quantile, with the white line in the middle denoting the median. The full antibody-virus matrix has 0.83 of measurements observed. (E) The landscape quantifies the tradeoffs in antibody potency and breadth, since an antibody strongly neutralizes nearby viruses and weakly neutralizes viruses further away. If the green and blue viruses are positioned in separate regions of the map, then the broad central antibody that moderately neutralizes all viruses will exhibit weaker neutralization than the more-specific antibodies near the green/blue viruses.

Figure S1C shows the antibody-virus dataset used to create the Neutralization Landscape in Figure 1C, where 83% of interactions are measured. To assess whether this number of measurements is sufficient to characterize the landscape, we withhold a random number of measurements, recreate the Neutralization Landscape, and compare the landscape predictions with the withheld values. Figure S1D shows that while the median of the resulting error distribution decreases slowly as more interactions are observed, the number of large outliers with $\langle \text{error} \rangle > 10$ drops drastically once at least 80% of values are observed. These results suggest that multidimensional scaling requires nearly-complete data to map a system.

Note that the solution of multidimensional scaling will not be unique (since translations, rotations, and reflections do not affect antibody-virus distances), but beyond these rigid transformations, there can be different configurations of antibodies and viruses with similar error. As discussed in Smith *et al.*, local minima can be avoided by starting the minimization algorithm at multiple different starting configurations and choosing the resulting map with the smallest error (*2*).

Applications and Limitations of the Neutralization Landscape

Given the widespread use of dimensionality reduction techniques (*e.g.*, multidimensional scaling, t-SNE, UMAP), it is worth emphasizing some applications of creating a framework that enumerates the full spectrum of antibody-virus responses (or some other biological context). These include:

- *Triangulating new antibodies/viruses*: Using ≈5 measurements for a new antibody or virus, we can fix its coordinate on the landscape and predict its full range of behavior (Figure 2). This approach can unify disparate datasets (*e.g.*, antibodies measured against viruses V_1-V_5 in one paper and V_6-V_{10} in another paper) by placing all of them on the "level playing field" presented by a single Neutralization Landscape.
- *Serum deconvolution:* A major challenge in immunology research is that experimentally characterizing the antibodies within serum requires months of effort, which hinders efforts to elicit a potent and durable antibody response. Our decomposition algorithm takes a step towards this goal, showing that it is computationally feasible for mixtures of 2-3 antibodies. When applied to sera, such an approach would quantify what fraction of antibodies target key viral epitopes (*e.g.*, the hemagglutinin head versus stem) and characterize when a response is polyclonal and hence more resilient to viral variants. Given that antibody fingerprinting has used monoclonal antibody data to decompose polyclonal HIV-1 sera, we hypothesize that our method should similarly generalize to the more complex case of serum decomposition provided that the antibodies within are sufficiently distinct (*5*).

- *Design a replicate for a serum sample:* Given that the antibody repertoire is constantly changing, a serum sample is an inherently limited commodity. Unlike with monoclonal antibodies, there is currently no method to make more of a serum of interest. By characterizing the dominant antibody signatures within a serum, we can rationally design an approximation for this serum using known antibodies. More precisely, we can subtract the neutralization of head antibodies and determine which stem antibodies on the map have the most similar neutralization profiles. Then, we can subtract the neutralization of those stem antibodies and determine which known head antibodies can give rise to the remaining signal. In this way, we can approximate all HA-targeting antibodies within a serum, and combine them with the appropriate stoichiometry to make the best possible approximation of the serum.
- *Antibody therapies and characterizing serum*: Given the increasing number of therapeutic antibodies under development (*6*, *7*), it is important to choose antibodies to cover as wide of a region on the landscape as possible with no holes and no redundancy. Similarly, groups seeking to understand serum responses should choose a diverse virus panel that covers a wide range of the map in order to detect as many distinct antibody signatures as possible.
- *Binding versus neutralization*: Although this work exclusively analyzes antibody neutralization, high-throughput assays can quantify antibody binding, providing complementary information on how well an antibody mixture binds to different viruses. It would be of great interest to compare landscapes created by binding versus neutralization measurements.
- *Degeneracy*: In this work, we found several instances where different antibody configurations can give rise to nearly identical neutralization profiles. This aspect of the immune response remains largely unexplored. How prevalent is this degeneracy, and how many different stem antibodies must be combined before the odds of correctly decomposing their collective response drops below 50% (see the fractions at the top of Figure 5E)?

In addition to these applications, we also mention some limitations of these dimensionality reduction approaches. First, dimensionality techniques characterize a given dataset, and it is not clear whether they can be extrapolated to characterize new behavior. While we demonstrate that a subset of our data can robustly predict the remainder of our measurements (Figure 2), additional viruses or antibodies may not conform to this same approach. Second, our assay only measured antibody neutralization, and hence remains ignorant of other mechanisms such as antibody-dependent cellular cytotoxicity or antibody-dependent cellular phagocytosis mediated through the Fc domain (*8*, *9*). Third, we only assessed H1N1 and H3N2 influenza viruses, and other subtypes could lie in vastly different regions of the landscape (or require a more complex metric or a higher dimension).

Finally, we emphasize one key difference between our approach (analyzing antibody-virus interactions) and traditional antigenic cartography (using serum-virus interactions), namely, that we do not assume any prior relationship between antibodies and viruses. Antigenic cartography assumes that serum elicited by infecting a ferret with virus *X* will be the maximally potent serum against virus *X* (more formally, that this serum is effectively a monoclonal antibody with the same coordinates as virus *X*). Thus, antigenic cartography cannot deal with asymmetries in inhibition data.

As a small but poignant example, consider the September 2019 WHO report (https://www.crick.ac.uk/sites/default/files/2019-10/CrickSH2019VCMreport_v2.pdf; page 31, Table 5-6,

first 2 rows and columns) assessing viruses V_1=H1N1 A/Michigan/45/2015 inhibited V_2=H1N1 A/Bayern/69/2009. Serum from a ferret infected with V_1 strongly inhibited V_1 (titer=640) but weakly inhibited V_2 (titer<40). With just this information, we would position the two viruses more than 4 units apart on an antigenic cartography map (where each unit corresponding to a 2-fold drop in HAI). However, a ferret infected with V_2 equally inhibited both V_1 and V_2 (titer=320); using the normalized antigenic cartography distance function, this serum must be at a distance=0 to both viruses, and hence both viruses must lie on the same point! This is a contradiction that cannot be resolved with higher-dimensional maps.

Our interpretation of these measurements is that the two viruses are antigenically distinct and should have different map positions, but the ferret infected with V_2 made a polyclonal response that inhibits both V_1 and V_2. This example demonstrates that both a normalized distance metric and a map based on polyclonal sera may distort the underlying structure of the landscape.

Computing the Error of a Neutralization Landscape

We applied multidimensional scaling to the monoclonal antibody neutralization data by minimizing the mean squared error between the predicted and measured IC_{50} values,

$$\langle \text{map error}^2 \rangle = 1/N \sum_{j\in\text{antibodies},k\in\text{viruses}} (d_{jk}^{\text{Predicted}} - d_{jk}^{\text{Measured}})^2 \, \Theta(d_{jk}^{\text{Predicted}}, d_{jk}^{\text{Measured}}),$$

using the number of measurements (N) and the predicted/measured antibody-virus map distances ($d_{jk}^{\text{Predicted}}/d_{jk}^{\text{Measured}}$) as described above. The function $\Theta(d_{jk}^{\text{Predicted}}, d_{jk}^{\text{Measured}})$ accounts for weak IC_{50} values above the dynamic range of our assay ($d_{jk}^{\text{Measured}} > 1.6\cdot 10^{-7}$ M, equivalent to 3.2 map units) so that the value of $d_{jk}^{\text{Predicted}}$ in such cases only contributes to the error when it falls below this bound. Following Smith *et al*., we define $\Theta(d_{jk}^{\text{Predicted}}, d_{jk}^{\text{Measured}})=1$ when an exact antibody-virus IC_{50} value is within the dynamic range of the assay ($d_{jk}^{\text{Measured}} \leq 3.2$), whereas $\Theta(d_{jk}^{\text{Predicted}}, d_{jk}^{\text{Measured}}) = \frac{1}{1+e^{-10\left(d_{jk}^{\text{Predicted}} - d_{jk}^{\text{Measured}}\right)}}$ for IC_{50}s outside our dynamic range ($d_{jk}^{\text{Measured}}>3.2$) (*2*).

To relate this error function to the more intuitive IC_{50} fold-error (equal to the ratio between the predicted and measured IC_{50} values or its inverse, whichever is ≥1), we convert the square root of this mean squared error on the map into IC_{50} ratios,

$$\langle \text{position error} \rangle \approx 10^{\sqrt{\langle \text{map error}^2 \rangle}}.$$

Note that mean-squared map error is the quantity minimized when creating the landscape, and the above relation demonstrates that minimizing the mean-squared map error will minimize the position error. However, once the MDS is complete, we compute the exact position error given by

$$\langle \text{position error} \rangle = 1/N \sum_{j\in\text{antibodies},k\in\text{viruses}} \text{fold-error}[IC_{50,jk}^{\text{predicted}}, IC_{50,jk}^{\text{measured}}]$$

where

$$\text{fold-error}[x,y] = \begin{cases} y/x, & x \leq y \\ x/y, & x > y. \end{cases}$$

This is the error we show on our 2D landscape (gray text in the bottom-right of Figure 1C). ⟨Position error⟩=1-fold implies that the landscape perfectly represents the data, while 2-fold error implies that the landscape IC_{50}s will be between 2-fold larger and 2-fold smaller than the measured values, on average.

Utilizing Monoclonal Antibodies to Create the HA Stem Neutralization Landscape

Previous work by Creanga *et al*. measured the neutralization IC_{50} of 18 HA stem-binding antibodies against 55 influenza strains (*10*). Replication-restricted reporter viruses (R3ΔPB1) were generated with

the PB1 gene replaced by a fluorescent protein. Neutralization IC_{50}s were inferred by titrating the concentration of antibodies, with the IC_{50} of each antibody-virus either fixed as the midpoint of the titration curve or bounded below as having $IC_{50} > 1.6 \cdot 10^{-7}$ M (25µg/mL).

In this work, we focus exclusively on the H1N1 and H3N2 strains in the Creanga virus panel (49/55), to which we added the more recent strains H1N1 A/Idaho/07/2018 and H3N2 A/Perth/1008/2019 [HA sequences available in this manuscript's GitHub repository]. We measured an additional 10 stem antibodies and excluded one of the original stem antibodies (315-53-1A07) that did not neutralize any of the viruses. 21 antibodies in our panel (named "315-xx-xxxx" or "xx-xxx") were isolated from volunteers vaccinated with an H7N9 vaccine in 2015 at the Vaccine Research Center (*11*, *12*). 58-6F03 is isolated from a volunteer vaccinated with an H5N1 vaccine in 2010 at the Vaccine Research Center (*13*, *14*). The six remaining antibodies were isolated between 2005-2015 from adults recently infected with influenza virus or vaccinated with seasonal influenza vaccine (CR6261, (*15*); CR8020 and CR9114, (*16*); CT149, (*17*); FI6v3, (*18*); MEDI8852, (*19*)), and hence their most recent H3N2 exposure was likely to an H3N2 around this time period (see Figure S2, blue H3N2 viruses #16-25 near the top of the H3N2 cluster).

The positions of these 51 viruses and 27 stem-binding antibodies are given in Table S1 as well as in the GitHub file "*(1) Neutralization Landscape Coordinates for the HA Stem.csv*". In addition to quantifying the total error of the 2D landscape (discussed in the previous section), we compute the error of each entry (*i.e.*, each antibody or virus) by quantifying how far it can move before its error is increased by 2-fold. For each entry, we compute the Hessian of $\langle \text{map error}^2 \rangle$ and find the direction $d_{\max}$ of maximal increase (and the perpendicular direction $d_{\min}$ where it minimally increases). We draw an ellipse whose semi-major axes ($r_{\max}$ and $r_{\min}$) in the maximal/minimal directions satisfied $\log_{10}(2) = \frac{1}{2}\, d_{\max/\min}\, r_{\max/\min}^2$, which corresponds to an ≈2-fold increase in map error. Across all antibodies and viruses, the uncertainty of all entries is always ≤0.4 units in any direction (Figure S2). We note that this uncertainty represents the *local* error of each antibody or virus about its map position, but there could be global minima elsewhere on the map where the entry would have similar error.

Comparison with other Dimensionality Reduction Methods

We assessed how our MDS algorithm performs against a few variant approaches. In each case, we a random 80% of the antibody-virus interactions (rather than the full 83% of measured data) to create the 2D landscape. We then used the landscape distance to infer the withheld measurements and calculated ⟨fold-error⟩ for these predictions.

We first compared our method against the established MDS approach from Smith *et al.*, where the primary difference in their approach is that the data from each antibody is first normalized to have distance=0 against the virus that it neutralizes most potently (*2*). Thus, we first divided each row in Figure S1C by $\min(IC_{50}\text{s})/(10^{-10}\text{ M})$ so that the smallest value in each row was 10^{-10} M (equivalent to zero distance with our metric). We then applied MDS, predicted the IC_{50} of each withheld interaction, multiplied these predictions by their appropriate row-factor (to return to absolute units), and compared against the measured value. This approach led to slightly worse predictions (⟨fold-error⟩=2.4±3.4 with normalization compared to ⟨fold-error⟩=2.3±2.6 with our approach), demonstrating that normalization leads to worse embeddings. This emphasizes that having antibody-virus measurements in absolute units, where every pair of antibody responses can be directly compared, leads to better characterizations of the system.

We next calculated the landscape using a semidefinite programming technique we recently developed that is well-suited to embed datasets with large outliers (by minimizing $\left|(d^{\text{Predicted}})^2-(d^{\text{Measured}})^2\right|$), although it performs worse on data with systematic noise (*20*). This method had substantially larger ⟨fold-error⟩=5.0±14.4, suggesting that this dataset has systematic noise and is not plagued with large outliers.

Lastly, we tried using the standard dimensionality reduction techniques such as PCA or UMAP, but we found them to be highly unsuitable because: (1) there is no distance metric, (2) they behave poorly with missing data, and (3) they are best suited to either characterizing the antibodies alone [using viruses as features] or the viruses alone [using the antibodies as features]. This pitfalls emphasize that our Neutralization Landscape is not simply a dimensionality reduction technique, but is rather a far more structured space where antibody-virus distance d directly corresponds to a neutralization measurement $IC_{50}=10^{-10+d}$ Molar.

As an aside, we note that when normalizing data, map distances are never "finalized." In the approach by Smith *et al*., the distance between virus i and antiserum j is given by $D_{ij}=b_j-\log_2(H_{ij})$, with $b_j=\log_2$ of the *maximum measurement* for antiserum j. Thus, if an additional virus is measured with a larger HAI titer against antiserum j, then all of j's other distances on the map would change. This potential pitfall is absent from our approach where IC_{50}s are not normalized but instead given in absolute units.

Visualizing Virus Evolution using Landscape Positions

By examining the positions of viruses across decades, we see markedly different patterns for H1N1 and H3N2 viruses. For each subtype, we color the viruses on the Neutralization Landscape in hues from lightest (oldest) to darkest (more recent strains). To focus on the antigenic evolution of the HA stem in humans, we exclude H1N1 A/New Jersey/8/1976 and H3N2 A/Indiana/10/2011 that crossed over from swine.

The H3N2 viruses tend to travel upwards along the landscape, moving at 0.022 units/year on average (Figure S2B, with H3N2 A/Aichi/2/1968 the most notable exception). To put that into context, after ~40 years, the antibodies below the H3N2 cluster would exhibit 10-fold less neutralization (since antibody-virus distance would increase by ≈1 unit). This HA stem evolution is markedly slower than the evolution of the highly-variable HA head (*21–23*), where hemagglutination inhibition decreases by 10-fold in ~10 years (approximately 2-fold per year) (*2*, *24*).

In contrast, the HA stem of H1N1 viruses is nearly stationary, moving upwards by only 0.003 units/year on average (Figure S2C, combining both the pre-pandemic [circles] and post-pandemic lineages [squares]). At this pace, it would take 300 years for an antibody below the H1N1 cluster to exhibit 10-fold less neutralization. These observations are corroborated by recent measurements showing that the human antibody response was minimally affected by H1N1 HA stem mutations from 2009-2019 (*25*).

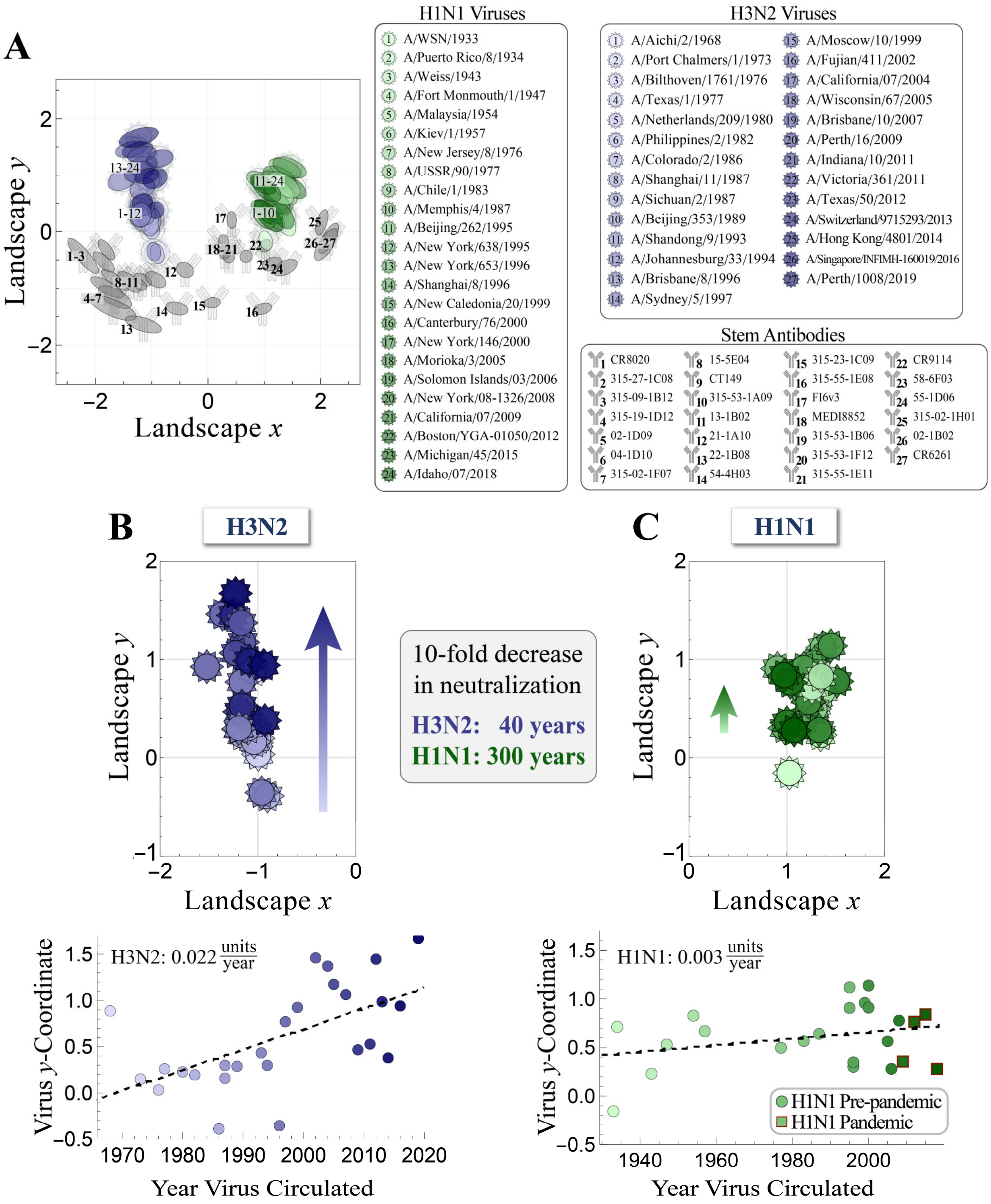

Supplementary Figure 2. Temporal patterns in virus neutralization. (A) The coordinates of each antibody and virus, overlaid with an ellipse showing the error of each coordinate (denoting a 2-fold increase in the entry's error). The antibody numbering used in this figure is followed throughout this work in all figures and tables, so that Stem Ab 1 represents CR8020, Stem Ab 2 represents 315-27-1C08, and so on. As in the main text, gridlines represent a 10x drop in neutralization. (B-C) *Top*, Close up of the H3N2 or H1N1 viruses on the landscape, showing that more recent H3N2 strains lie towards the top of the landscape. *Bottom*, *y*-coordinate for each virus versus its year of circulation, together with the best fit line.

Supplementary Table 1. Coordinates of the viruses and antibodies (Abs) on the Neutralization Landscape. For each entry, we specify its map coordinates along with the size of the semi-major and semi-minor axis of the uncertainty ellipse shown in Figure S2A (subscript θ given in radians counterclockwise from the *x*-axis). The head antibodies used in some of the antibody mixtures are listed, although they are not positioned on the stem landscape.

Virus or Antibody	Coordinate	Large Error (Angle)	Small Error (Angle)
H1N1 A/WSN/1933	(1.03, -0.16)	0.34 (θ=2.05)	0.17 (θ=0.48)
H1N1 A/Puerto Rico/8/1934	(1.26, 0.71)	0.24 (θ=2.35)	0.14 (θ=0.78)
H1N1 A/Weiss/1943	(1.34, 0.23)	0.25 (θ=2.47)	0.15 (θ=0.89)
H1N1 A/Fort Monmouth/1/1947	(1.3, 0.53)	0.24 (θ=2.11)	0.12 (θ=0.54)
H1N1 A/Malaysia/1954	(1.35, 0.83)	0.24 (θ=2.31)	0.14 (θ=0.74)
H1N1 A/Kiev/1/1957	(1.37, 0.67)	0.23 (θ=2.36)	0.14 (θ=0.79)
H1N1 A/New Jersey/8/1976	(1.2, 0.23)	0.23 (θ=2.45)	0.14 (θ=0.88)
H1N1 A/USSR/90/1977	(1.28, 0.5)	0.3 (θ=2.75)	0.15 (θ=1.18)
H1N1 A/Chile/1/1983	(1.23, 0.57)	0.24 (θ=2.64)	0.14 (θ=1.07)
H1N1 A/Memphis/4/1987	(1.19, 0.64)	0.3 (θ=2.17)	0.16 (θ=0.6)
H1N1 A/Beijing/262/1995	(1.37, 1.12)	0.24 (θ=2.17)	0.13 (θ=0.6)
H1N1 A/New York/638/1995	(0.9, 0.91)	0.32 (θ=2.69)	0.17 (θ=1.11)
H1N1 A/New York/653/1996	(0.98, 0.3)	0.25 (θ=2.64)	0.14 (θ=1.07)
H1N1 A/Shanghai/8/1996	(1.15, 0.35)	0.32 (θ=2.75)	0.15 (θ=1.18)
H1N1 A/New Caledonia/20/1999	(1.27, 0.96)	0.29 (θ=2.3)	0.17 (θ=0.73)
H1N1 A/Canterbury/76/2000	(1.17, 0.91)	0.26 (θ=2.18)	0.16 (θ=0.61)
H1N1 A/New York/146/2000	(1.45, 1.14)	0.26 (θ=2.52)	0.15 (θ=0.94)
H1N1 A/Morioka/3/2005	(1.22, 0.56)	0.25 (θ=2.18)	0.12 (θ=0.61)
H1N1 A/Solomon Islands/03/2006	(1.33, 0.28)	0.34 (θ=2.27)	0.17 (θ=0.7)
H1N1 A/New York/08-1326/2008	(1.51, 0.78)	0.18 (θ=1.9)	0.11 (θ=0.33)
H1N1 A/California/07/2009	(1., 0.36)	0.31 (θ=2.47)	0.18 (θ=0.9)
H1N1 A/Boston/YGA-01050/2012	(1.04, 0.76)	0.32 (θ=2.55)	0.18 (θ=0.98)
H1N1 A/Michigan/45/2015	(0.97, 0.84)	0.31 (θ=2.44)	0.16 (θ=0.87)
H1N1 A/Idaho/07/2018	(1.07, 0.28)	0.25 (θ=2.57)	0.14 (θ=1.)
H3N2 A/Aichi/2/1968	(-1.05, 0.89)	0.2 (θ=0.27)	0.13 (θ=1.84)
H3N2 A/Port Chalmers/1/1973	(-1.01, 0.15)	0.23 (θ=1.63)	0.16 (θ=0.06)
H3N2 A/Bilthoven/1761/1976	(-1., 0.03)	0.16 (θ=1.65)	0.14 (θ=0.08)
H3N2 A/Texas/1/1977	(-1.01, 0.26)	0.15 (θ=0.86)	0.14 (θ=2.43)
H3N2 A/Netherlands/209/1980	(-1.16, 0.23)	0.22 (θ=1.53)	0.18 (θ=3.1)
H3N2 A/Philippines/2/1982	(-1., 0.19)	0.23 (θ=1.59)	0.16 (θ=0.02)
H3N2 A/Colorado/2/1986	(-0.9, -0.39)	0.18 (θ=1.81)	0.12 (θ=0.24)
H3N2 A/Shanghai/11/1987	(-1.04, 0.16)	0.24 (θ=1.62)	0.16 (θ=0.04)
H3N2 A/Sichuan/2/1987	(-1.1, 0.3)	0.19 (θ=1.13)	0.16 (θ=2.7)

H3N2 A/Beijing/353/1989	(-1.2, 0.29)	0.22 (θ=1.39)	0.19 (θ=2.96)
H3N2 A/Shandong/9/1993	(-1.12, 0.43)	0.17 (θ=0.41)	0.14 (θ=1.98)
H3N2 A/Johannesburg/33/1994	(-1.19, 0.3)	0.17 (θ=1.71)	0.12 (θ=0.14)
H3N2 A/Brisbane/8/1996	(-0.96, -0.36)	0.23 (θ=0.47)	0.17 (θ=2.04)
H3N2 A/Sydney/5/1997	(-1.17, 0.77)	0.27 (θ=0.46)	0.18 (θ=2.03)
H3N2 A/Moscow/10/1999	(-1.52, 0.93)	0.29 (θ=0.24)	0.13 (θ=1.81)
H3N2 A/Fujian/411/2002	(-1.36, 1.46)	0.26 (θ=0.28)	0.16 (θ=1.85)
H3N2 A/California/07/2004	(-1.17, 1.37)	0.27 (θ=0.31)	0.18 (θ=1.88)
H3N2 A/Wisconsin/67/2005	(-1.15, 1.18)	0.21 (θ=0.78)	0.19 (θ=2.35)
H3N2 A/Brisbane/10/2007	(-1.23, 1.06)	0.18 (θ=0.35)	0.14 (θ=1.92)
H3N2 A/Perth/16/2009	(-1.19, 0.47)	0.28 (θ=0.21)	0.13 (θ=1.78)
H3N2 A/Indiana/10/2011	(-0.86, 1.29)	0.22 (θ=0.25)	0.13 (θ=1.82)
H3N2 A/Victoria/361/2011	(-1.16, 0.53)	0.15 (θ=0.88)	0.15 (θ=2.45)
H3N2 A/Texas/50/2012	(-1.25, 1.45)	0.22 (θ=0.43)	0.17 (θ=2.)
H3N2 A/Switzerland/9715293/2013	(-1.09, 0.99)	0.36 (θ=0.3)	0.15 (θ=1.87)
H3N2 A/Hong Kong/4801/2014	(-0.93, 0.38)	0.22 (θ=1.46)	0.19 (θ=3.03)
H3N2 A/Singapore/INFIMH-160019/2016	(-0.94, 0.94)	0.26 (θ=0.2)	0.13 (θ=1.77)
H3N2 A/Perth/1008/2019	(-1.23, 1.67)	0.24 (θ=0.24)	0.15 (θ=1.81)
Stem Ab 1 (CR8020)	(-2.22, -0.43)	0.4 (θ=2.27)	0.11 (θ=0.7)
Stem Ab 2 (315-27-1C08)	(-1.85, -0.54)	0.24 (θ=2.38)	0.1 (θ=0.81)
Stem Ab 3 (315-09-1B12)	(-1.81, -0.32)	0.21 (θ=2.21)	0.09 (θ=0.64)
Stem Ab 4 (315-19-1D12)	(-1.79, -0.98)	0.33 (θ=2.64)	0.11 (θ=1.07)
Stem Ab 5 (02-1D09)	(-1.65, -1.16)	0.33 (θ=2.63)	0.14 (θ=1.06)
Stem Ab 6 (04-1D10)	(-1.69, -1.35)	0.44 (θ=2.74)	0.15 (θ=1.17)
Stem Ab 7 (315-02-1F07)	(-1.65, -0.86)	0.29 (θ=2.53)	0.1 (θ=0.96)
Stem Ab 8 (15-5E04)	(-1.4, -0.89)	0.25 (θ=2.46)	0.12 (θ=0.89)
Stem Ab 9 (CT149)	(-1.24, -0.92)	0.16 (θ=2.61)	0.09 (θ=1.04)
Stem Ab 10 (315-53-1A09)	(-1.16, -0.85)	0.17 (θ=2.65)	0.09 (θ=1.08)
Stem Ab 11 (13-1B02)	(-0.93, -0.85)	0.2 (θ=2.61)	0.12 (θ=1.04)
Stem Ab 12 (21-1A10)	(-0.41, -0.68)	0.16 (θ=2.6)	0.13 (θ=1.03)
Stem Ab 13 (22-1B08)	(-1.15, -1.64)	0.33 (θ=2.85)	0.12 (θ=1.28)
Stem Ab 14 (54-4H03)	(-0.55, -1.36)	0.2 (θ=2.92)	0.11 (θ=1.35)
Stem Ab 15 (315-23-1C09)	(0.08, -1.26)	0.14 (θ=0.09)	0.09 (θ=1.66)
Stem Ab 16 (315-55-1E08)	(0.97, -1.36)	0.17 (θ=0.3)	0.09 (θ=1.87)
Stem Ab 17 (FI6v3)	(0.42, 0.21)	0.15 (θ=1.63)	0.08 (θ=0.06)
Stem Ab 18 (MEDI8852)	(0.28, -0.19)	0.13 (θ=1.68)	0.09 (θ=0.11)
Stem Ab 19 (315-53-1B06)	(0.3, -0.37)	0.12 (θ=1.63)	0.1 (θ=0.06)

Stem Ab 20 (315-53-1F12)	(0.37, -0.44)	0.11 (θ=1.46)	0.1 (θ=3.03)
Stem Ab 21 (315-55-1E11)	(0.68, -0.44)	0.11 (θ=0.92)	0.11 (θ=2.49)
Stem Ab 22 (CR9114)	(1.02, -0.25)	0.12 (θ=0.67)	0.1 (θ=2.24)
Stem Ab 23 (58-6F03)	(1.13, -0.57)	0.18 (θ=0.39)	0.11 (θ=1.97)
Stem Ab 24 (55-1D06)	(1.37, -0.64)	0.23 (θ=0.45)	0.12 (θ=2.03)
Stem Ab 25 (315-02-1H01)	(2.02, 0.16)	0.23 (θ=1.28)	0.09 (θ=2.85)
Stem Ab 26 (02-1B02)	(2.1, -0.23)	0.32 (θ=0.99)	0.13 (θ=2.56)
Stem Ab 27 (CR6261)	(2.14, -0.12)	0.3 (θ=1.05)	0.11 (θ=2.62)
Head Ab 1 (C05)	–	–	–
Head Ab 2 (CH65)	–	–	–
Head Ab 3 (F005-126)	–	–	–
Head Ab 4 (F045-092)	–	–	–
Head Ab 5 (310-33-1G06)	–	–	–
Head Ab 6 (5J8)	–	–	–

This stark difference between the H1N1 and H3N2 viruses may be caused by a greater tolerance for HA stem mutations in H3N2 viruses (*26*, *27*). It is known that H3N2 viruses acquire more mutations per year (*28*), and consequently, the H3N2 component of the influenza vaccine changed 8 times in the past decade, whereas the H1N1 component only changed 4 times. Biologically, the strong upwards trend of the H3N2 strains could represent virus evolution spurred by the HA-stem antibody response. While we note that broadly neutralizing anti-HA stem antibodies are rare in the human antibody repertoire, the majority of the antibodies on our panel are positioned below the viruses, which could drive them upwards (*12*). Moreover, the broadest neutralizing antibodies (*e.g.*, FI6v3, CR9114, MEDI8852, and 315-53-1B06) lie closer to the H1N1 viruses than to the H3N2 viruses consistent with previous findings that they evolved from unmutated common ancestors able to bind only group 1 HAs (*e.g.*, H1, H2, or H5) and acquired the ability to neutralize H3N2 viruses through somatic hypermutation (*12*, *18*, *19*, *29*, *30*).

As an aside, when we looked at every pair of H3N2 viruses, we found a weak correlation between the map distance and sequence distance [the edit distance between the HA stem sequences] of r^2=0.1, but no correlation for the H1N1 viruses, r^2=0.

Dimensionality of the Landscape

Since the Neutralization Landscape attempts to quantify the complex and potentially highly-multidimensional antibody-virus interactions in 2D, the position error could be greatly reduced in higher dimensional representations. To explore this possibility, we recreated the landscape from Figure 1C in 1D, 2D, and 3D (Figure S3).

We quantify how accurately these maps represent the data in two separate ways. First, we compute the position error, given as the fold-error between the mapped IC_{50} and measured IC_{50}, averaged over every antibody-virus pair (see the section *Computing the Error of a Neutralization Landscape* above). For example, if an antibody-virus pair has a predicted $IC_{50}=10^{-9}$ M and a measured $IC_{50}=2\cdot10^{-9}$ M, it has a fold-error of 2. By analyzing all antibody-virus pairs (shown by the row of plots with gold points in

Figure S3), we find that the position error decreases from 10.6-fold (1D) to 2.2-fold (2D) to 1.9-fold (3D). Thus, a 1D map does not have enough flexibility to characterize these antibody-virus interactions, whereas maps in two-or-more dimensions have an average fold-error on par with experimental noise. Indeed, going into higher dimensions may overfit the data if it has a simpler structure.

To test for potential overfitting, we assessed each map using the leave-one-out procedure described in Figure 2. Briefly, we withheld an antibody or virus, recreated the map, triangulated the withheld entity using 6 measurements, and then predicted all remaining measurements (Figure S3, bottom row of plots with purple points). Averaged over all predictions, the 2D representation yielded the best predictions (Figure S3A, purple line), demonstrating that the higher dimensional representations somewhat overfit the data. Taken together, these results suggest that this dataset is two-dimensional, as has been commonly observed in other applications of antigenic cartography (*24*).

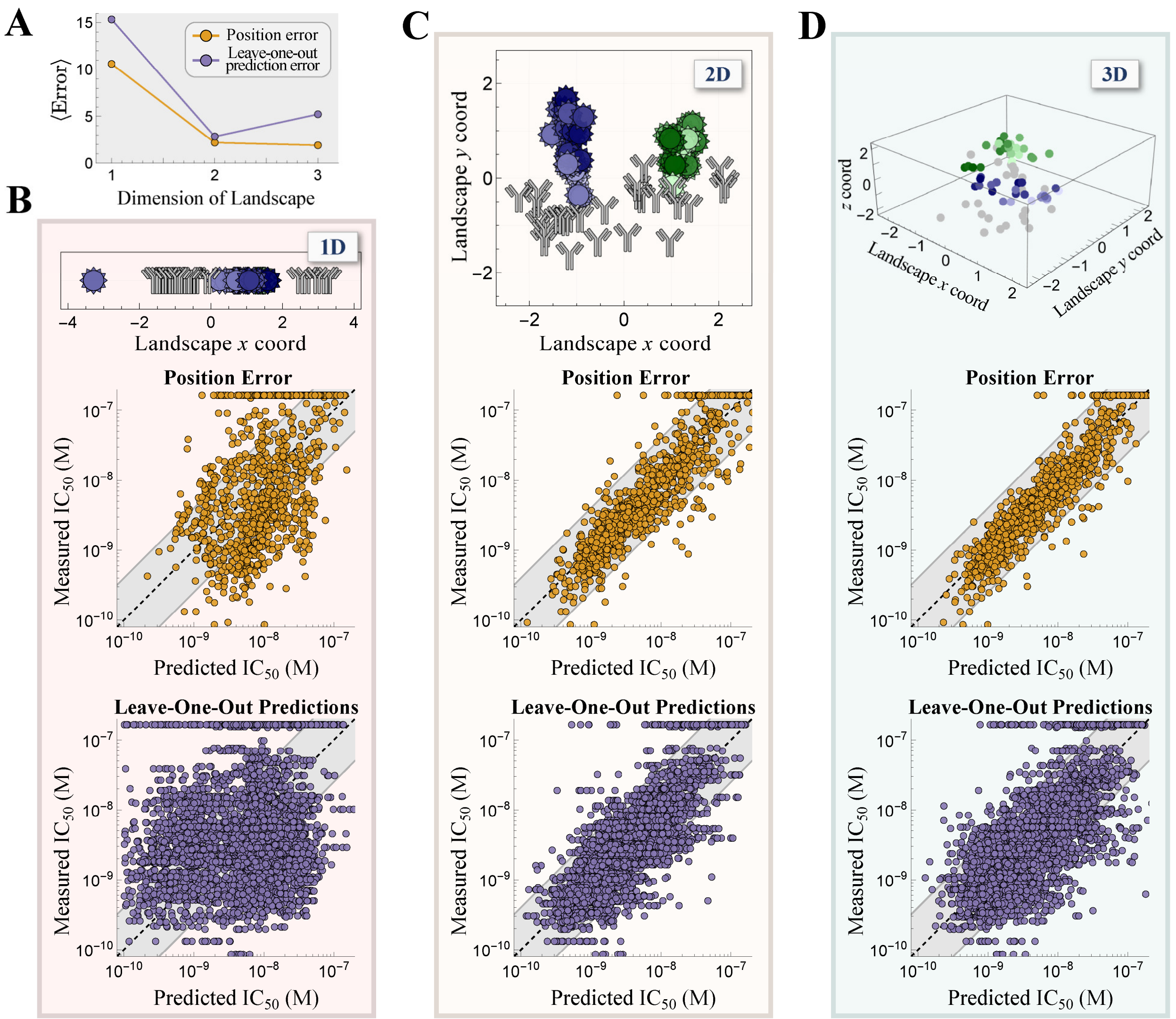

Supplementary Figure 3. Dimensionality of the Neutralization Landscape. Monoclonal antibody neutralization data was combined with multidimensional scaling in 1D, 2D, and 3D to create antigenic maps of the virus strains. (A) In each dimension, we compute how accurately the map represents the data through its position error (average of each fold-error between every measured and mapped IC_{50}; gold points). We further quantify the predictive power through leave-one-out analysis, where an antibody or virus is withheld, triangulated using 6 measurements, and its other interactions are predicted (purple). We show the resulting maps in (B) 1D, (C) 2D, and (D) 3D along with plots

showing the individual IC_{50} predictions used to calculate the position error (gold) and leave-one-out predictions (purple). In each plot, gray band represents ≤4-fold error, where 1-fold error represents an exact prediction.

Extrapolating the Behavior of New Antibodies

Using the positions of the antibodies and viruses on the Neutralization Landscape, we can add additional entries using a few measurements to triangulate their coordinates (Figure 2). Although triangulation using 3 entries would work with perfect data, we need more measurements to account for noise and for the limited dynamic range of the experiment (*i.e.*, the bound $IC_{50} > 25\,\mu g/mL$ gives much more limited information than an exact value such as $IC_{50} = 10\,\mu g/mL$). Since some antibodies only neutralized H1N1 viruses while others only neutralized H3N2 viruses, we always chose N=6 viruses (3 H1N1 + 3 H3N2) for antibody triangulation.

Moreover, triangulation works slightly better when the entries used for triangulation are spread apart on the map, since entries that are close together provide the same information. For example, since the H1N1 and H3N2 viruses lie on approximately vertical lines, we chose the 3 H1N1 viruses and 3 H3N2 viruses to be spread out based on their y-coordinates (see the middle panel of Figure 2A). Similarly, when triangulating viruses we chose the antibodies to be spread apart based on their x-coordinate (since the antibodies lie on an approximately horizontal line).

When comparing the predicted versus measured neutralization, we treat the bounded measurements (IC_{50}>25μg/mL) carefully. We choose to adopt the harshest possible error penalty, including a bounded measurement when we predict it *incorrectly*. For example, if we predict an IC_{50}=20μg/mL for a measurement of >25μg/mL, we draw this prediction at the point (20μg/mL,25μg/mL) [see the points along the top edge of Figure 2B]. On the other hand, if we predict a value such as IC_{50}=30μg/mL for a measurement of >25μg/mL, we do not include that point in our plot to prevent deflating the error.

As an edge case, we note that we only consider an antibody to be triangulated relative to the H1N1 or H3N2 subtype if at least one of its triangulating IC_{50} values lies within the dynamic range of the experiment. In the rare cases where the 3 H1N1 viruses used to triangulate an antibody all had IC_{50}s>25μg/mL, we excluded all predictions against every H1N1 virus since there is not enough information to precisely pinpoint its location (although we still include its predictions against H3N2 viruses). This same rule applies when we replace H1N1↔H3N2.

Flagging Potential Outliers in a Dataset

As described in the main text, one application of the Neutralization Landscape is to identify antibody-virus measurements that do not conform with the underlying structure of the map. When we first constructed the landscape, we found that 16/1148 (≈1%) of measurements were represented by an IC_{50} on the landscape that was >10-fold off from the measurement. When we remeasured all 16 interactions, we found that in 13/16 cases the new measurement had <10-fold error, and in particular this held for the three largest outliers with >40-fold error (Figure S4A).

We further remeasured 54 non-outlier interactions with <10-fold error. Although in a single case the remeasured value became a large outlier, in the other 53/54 cases the remeasured value was <10-fold apart from the landscape IC50 value. These results show that the Neutralization Landscape can be used to flag potential outliers, an important application in datasets where self-consistency is often hard to determine directly from the measurements.

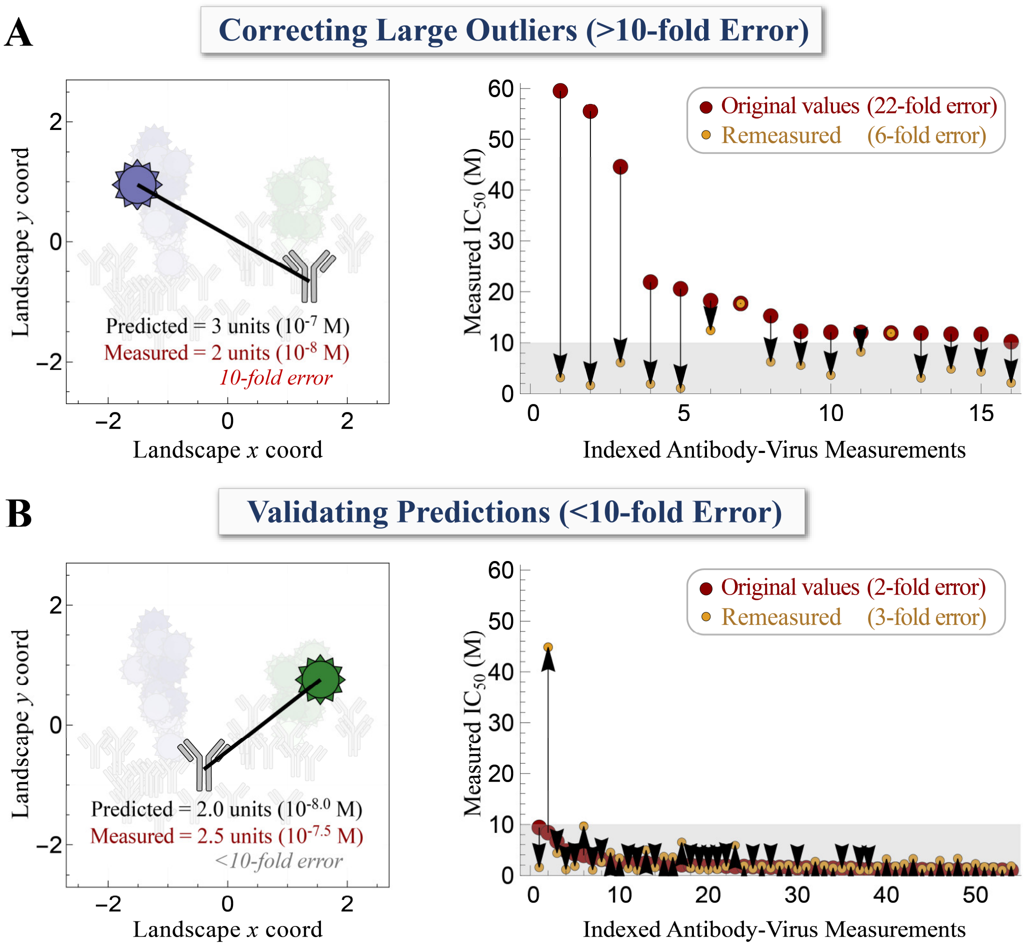

Supplementary Figure 4. Identifying and correcting outliers in the dataset. (A) *Left*, We identified antibody-virus measurements that were >10-fold off from the IC_{50} predicted by the landscape. *Right*, The 16 largest outliers [red points] were remeasured [gold points]. (B) We similarly remeasured 54 antibody-virus interactions whose measurements were within 10-fold of the prediction to ensure that we could separate outliers from true measurements. Aside from one inexplicably large outlier (remeasuring H1N1 A/California/07/2009 vs antibody 21-1A10 changed the IC_{50} from 4.7μg/mL→>25μg/mL), the other 53/54 measurements had both original and remeasured fold-error <10.

Adding New Antibodies or Viruses to the Map

We use neutralization data from Table 1 of Nakamura *et al*. to demonstrate how new antibodies can be added to the map (and new viruses can be similarly added) (*31*). The authors measured the response of four antibodies (called 39.18, 39.29, 81.39, and 36.89) against five H1N1 viruses and eight H3N2 viruses, enabling us to triangulate the locations of each antibody by using the virus coordinates in Table S1.

As a minor technical note, when a virus in this dataset is not on our virus panel, we can either associate it with the most similar virus (*i.e.*, a virus with a similar sequence) or drop the virus. For example, of the five H1N1 viruses (A/California/07/2009, A/Brisbane/59/2007, A/Solomon Islands/03/2006, H1N1 A/New Caledonia/20/1999, and A/Puerto Rico/8/1934; see Figure S5B) we substituted A/Brisbane/59/2007→A/New York/08-1326/2008 [which according to GISAID consensus sequences differ by ΔAA=2 amino acids, both of which are in the HA head], while the other four H1N1 were in our panel. Moreover, of the eight H3N2 viruses (A/Victoria/361/2011, A/Perth/16/2009, A/Brisbane/10/2007, A/Wisconsin/67/2005, A/Victoria/3/1975, A/Port Chalmers/1/1973, A/Hong Kong/8/1968,

A/Aichi/2/1968), we substituted A/Victoria/3/1975→A/Bilthoven/1761/1976 [ΔAA=5, with all mutations in the HA head], dropped A/Hong Kong/8/1968 [for which we had no sequence], and had exact matches with all remaining viruses. (While matching viruses in this manner enables us to utilize more data, it assumes that viruses with similar sequences will have similar neutralization profiles. Both viruses we substituted only had mutations in the HA head, which we expect will minimally affect the neutralization of stem-targeting antibodies. However, an open question is how similar two viruses must be before this matching technique breaks down. While the two datasets analyzed in this work are too small to rigorously assess this question, this question could be determined for larger dataset by assessing the accuracy of the triangulation calculations described below when more distant viruses are matched.)

After matching the viruses, we triangulated the antibody positions using all measurements to visually confirm that the IC_{50}s pointed to a single location on the map (Figure S5A). All four antibodies were consistent with the structure of the map, lying near all the virus measurements [red circles] as well as lying outside the zones of exclusion [gold disks]. Two antibodies (39.29 and 81.39) are positioned slightly above the midpoint of the H1N1 and H3N2 viruses, suggesting that they are near the optimal phenotype to neutralize both subtypes. More precisely, the predicted optimal antibody against these viruses [analogous to the purple antibody in Figure 3B, albeit for a different set of viruses] would have an $IC_{50}^{min} \leq 24 \cdot 10^{-10}$ M, whereas antibody 81.39 achieves an $IC_{50}^{panel} \leq 146 \cdot 10^{-10}$ M, only 6-fold higher (Figure S5B).

As further validation, we assessed how well each antibody could be triangulated by 6 measurements in order to predict the other 6 values. To triangulate, we used the three H1N1 (A/California/07/2009, A/Solomon Islands/03/2006, A/Puerto Rico/8/1934) and three H3N2 viruses (A/Wisconsin/67/2005, A/Port Chalmers/1/1973, A/Aichi/2/1968) that were widely spread apart on the map. The resulting predictions are shown in graphical and table form, where we have plotted neutralization of $>16{,}000 \cdot 10^{-10}$ M as this lower bound (Figure S5B). On average, we find that the withheld measurements are within 2.6-fold of the predictions, confirming our predictive power. Having confirmed this predictive power, we then returned to the triangulations shown in Figure S5A using all 12 measurements, and with those antibody positions we predicted the antibody neutralization against the remaining 36 viruses on our panel (Figure S5C).

The Triangle Inequality, Antibody-Virus Distance as a Metric

For antibody-virus distance to form a metric, it must obey a triangle inequality. Colloquially, the triangle inequality ensures that the shortest distance between two points is a straight line, and that a more circuitous path does not represent a shortcut.

Given that antibody-virus distance corresponds to an experimentally measurable neutralization measurement (whereas antibody-antibody or virus-virus distance cannot be directly measured), we modify the usual triangle inequality to include two antibodies and two viruses (Figure S6, Equation 1). Although this relationship would more accurately be called a quadrilateral inequality, we will continue to refer to it as the more familiar triangle inequality. By analyzing all combinations of antibodies and viruses, we perform 400,000 tests of the triangle inequality, and we find that 98.8% satisfy Equation 1. If we account for the 2-fold error of the neutralization assay, the fraction increases to 99.7%, demonstrating that the triangle inequality is overwhelmingly satisfied on our antibody-virus data.

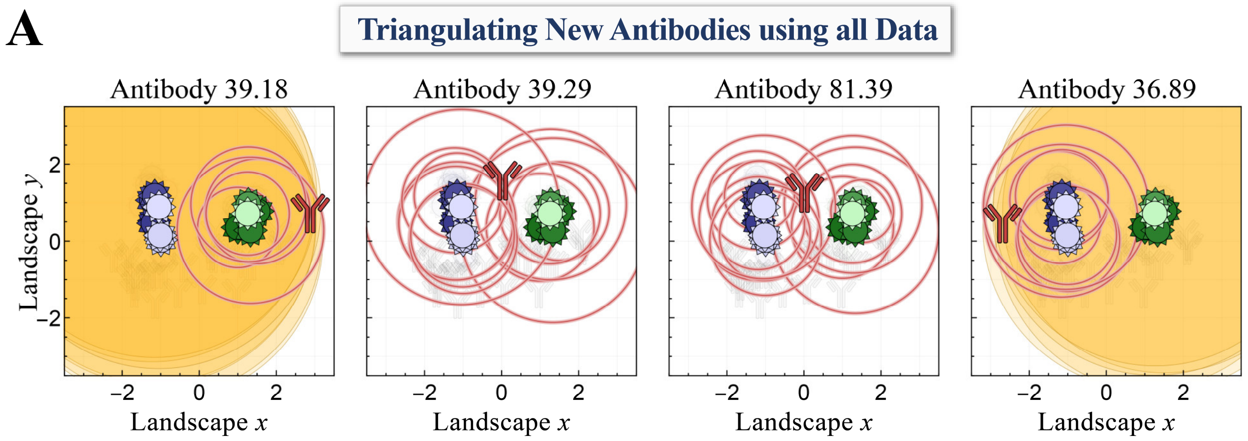

B

Predictions using a Subset of Data

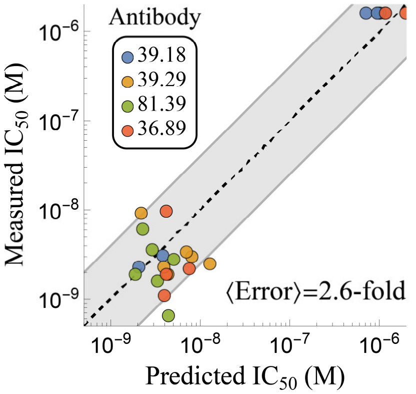

Data to Triangulate each Antibody

IC_{50} (× 10^{-10} Molar)	Ab 39.18	Ab 39.29	Ab 81.39	Ab 36.89
H1N1 A/California/07/2009	11	25	21	>16 000
H1N1 A/Solomon Islands/03/2006	80	251	146	>16 000
H1N1 A/Puerto Rico/8/1934	12	20	19	>16 000
H3N2 A/Wisconsin/67/2005	>16 000	13	8.1	16
H3N2 A/Port Chalmers/1/1973	>16 000	22	15	19
H3N2 A/Aichi/2/1968	>16 000	350	73	139

Comparing Predictions with Remaining Data

IC_{50} (× 10^{-10} Molar)	Ab 39.18	Ab 39.29	Ab 81.39	Ab 36.89
H1N1 A/New Caledonia/20/1999	31	92	61	>16 000
	38	22	23	11 700
H1N1 A/New York/08−1326/2008	23	19	6.5	>16 000
	21	43	44	19 800
H3N2 A/Bilthoven/1761/1976	>16 000	25	28	22
	7100	130	50	75
H3N2 A/Brisbane/10/2007	>16 000	23	19	19
	11 600	39	19	42
H3N2 A/Perth/16/2009	>16 000	30	16	11
	10 100	80	33	40
H3N2 A/Victoria/361/2011	>16 000	34	36	97
	9500	70	29	42

C

Predicting Full Antibody Response

Predictions across the Full Virus Panel

IC_{50} (× 10^{-10} Molar)	Ab 39.18	Ab 39.29	Ab 81.39	Ab 36.89
H1N1 A/WSN/1933	110	100	54	5900
H1N1 A/Weiss/1943	41	76	48	11 100
H1N1 A/Fort Monmouth/1/1947	39	44	30	9800
H1N1 A/Malaysia/1954	34	33	25	11 500
H1N1 A/Kiev/1/1957	33	41	30	11 600
H1N1 A/New Jersey/8/1976	56	60	37	7900
H1N1 A/USSR/90/1977	41	45	30	9500
H1N1 A/Chile/1/1983	45	37	25	8500
H1N1 A/Memphis/4/1987	49	31	21	7700
H1N1 A/Beijing/262/1995	37	26	23	13 000
H1N1 A/New York/638/1995	97	13	9	4200
H1N1 A/New York/653/1996	89	39	23	4700
H1N1 A/Shanghai/8/1996	59	46	29	7000
H1N1 A/Canterbury/76/2000	52	21	16	7800
H1N1 A/New York/146/2000	31	31	28	15 800
H1N1 A/Morioka/3/2005	47	37	24	8200
H1N1 A/Boston/.../2012	70	20	14	5500
H1N1 A/Michigan/45/2015	82	16	11	4900
H1N1 A/Idaho/07/2018	73	46	28	5900
H3N2 A/Netherlands/209/1980	11 800	62	36	35
H3N2 A/Philippines/2/1982	8200	52	29	52
H3N2 A/Colorado/2/1986	8800	150	76	96
H3N2 A/Shanghai/11/1987	9100	59	33	48
H3N2 A/Sichuan/2/1987	10 100	51	29	40
H3N2 A/Beijing/353/1989	12 700	60	36	32
H3N2 A/Shangdong/9/1993	10 200	41	25	38
H3N2 A/Johannesburg/33/1994	12 500	58	35	32
H3N2 A/Brisbane/8/1996	9800	150	76	82
H3N2 A/Moscow/10/1999	25 600	48	37	18
H3N2 A/Fujian/411/2002	20 300	25	25	46
H3N2 A/California/07/2004	12 700	16	16	59
H3N2 A/Indiana/10/2011	6100	8	7	100
H3N2 A/Switzerland/.../2013	9500	18	14	49
H3N2 A/Hong Kong/4801/2014	6700	34	19	58
H3N2 A/Singapore/.../2016	6700	14	10	66
H3N2 A/Perth/1008/2019	16 500	18	20	80

Supplementary Figure 5. Adding antibodies to the map from an external dataset. (A) Triangulating four antibodies from Nakamura *et al*. using all 12 measurements of each antibody (18). (B) Confirming the predictive power of the landscape by using six measurements for each antibody [top six rows, blue text] to predict the remaining six values [bottom six rows, predictions in pink]. Gray band in the plot represents ≤4-fold error, where 1-fold error represents an exact prediction. (C) Predictions against the remaining 36 viruses in our panel using all 12 measurements for each antibody.

Removing Head Antibody Neutralization

Although virus-virus distance between viruses V1 and V2 cannot be directly measured, it nonetheless constrains the fold-difference in neutralization measurements (Figure 4B). For example, if two viruses lie near each other on the landscape ($d_{\text{V1-V2}} \approx 0$), then any stem antibody will have nearly identical distance to both viruses. Hence its neutralization should also be identical, resulting in a fold-difference≈1 between the antibody's IC_{50}s against both viruses; in the limit $d_{\text{V1-V2}}=0$, we achieve a fold-difference=1 against all possible viruses.

If we consider all pairs of viruses within a distance $d_{\text{V1-V2}}=0.3$ of one another, their neutralization against any virus should differ by $10^{0.3}$=2-fold (Figure S6B), and hence with 2-fold experimental error we expect that their IC_{50}s should differ by no more than 4-fold. In our dataset, 161 pairs of viruses lie within 0.3 units of each other, which collectively have 2216 IC50s measured against our antibody panel. Of these, 65% of paired IC_{50}s are within 2-fold and 90% are within 4-fold of each other, in line with our expectation of experimental error.

As another example, if two viruses are 2 units apart, their IC_{50}s can differ by at most 100-fold (Figure S6C), with this maximum achieved when an antibody is collinear with the two viruses but does not lie between them (*e.g.*, brown antibody on the left). Antibodies lying on the perpendicular bisector to the segment connecting the two viruses will equally neutralize both viruses and hence achieve the minimum possible fold-difference of 1 (*e.g.*, pink antibody in the center).

When removing the neutralization of head antibodies, we use the constraint imposed by virus-virus distance. For example, if two viruses are $d_{\text{V1-V2}}$=2 units apart as in Figure S6C, and if a head+stem mixture exhibits an $\text{IC}_{50,\text{V1}}^{\text{mixture}} = 10^{-11}\text{M}$ and $\text{IC}_{50,\text{V2}}^{\text{mixture}} = 10^{-7}\text{M}$, then the stem antibody alone is bounded below by $\text{IC}_{50,\text{V1}}^{\text{stem Ab}} \geq \text{IC}_{50,\text{V2}}^{\text{mixture}} 10^{-d_{\text{V1-V2}}}\text{M} = 10^{-9}\text{M}$ (always using the larger $\text{IC}_{50}^{\text{mixture}}$ value), since the two measurements can be at most $10^{d_{\text{V1-V2}}}$ =100-fold different and both the stem and head antibodies weakly neutralize V2. However, this does not account for the noise in the assay, and hence we take the more conservative approach and only increase an IC_{50} if it is more than f_{noise}=10-fold lower than this lower bound. In the example above, we would set $\text{IC}_{50,\text{V1}}^{\text{stem Ab}} \geq \frac{\text{IC}_{50,\text{V2}}^{\text{mixture}}}{f_{\text{noise}} 10^{d_{\text{V1-V2}}}\text{M}} = 10^{-10}\text{M}$ to account for noise.

Note that we always use the larger mixture IC_{50} to bound the stem antibody's neutralization. If a head+ stem mixture exhibits a large IC_{50} against a virus, then both the head and stem antibodies must be weak against that virus. However, if this mixture exhibits a small IC_{50}, either the head antibody or stem antibody (or both) could potently neutralize the virus, and in the former case we should not use the mixture's IC_{50} to alter any other values. Thus, we always use the larger mixture IC_{50} values as "ground truth" to subtract the head antibody signal.

On the landscape, we visualize the new IC_{50} bounded from below as a "zone of exclusion" represented by a gold disk, so that the stem antibody should lie as close to the red circles [signifying an IC_{50} within the dynamic range of the assay] and outside all gold disks [representing IC_{50}s bounded from below] (Figure 4C). For example, when removing the head antibody signatures in Figure 4C, the red circles around the blue H3N2 viruses (left panel) expanded due to the constraint on virus-virus distance and became lower bounds (shown by gold circles in the right panel). The IC_{50}s against the green H1N1 viruses did not

change because they all obeyed the constraints of the neutralization landscape. This (correctly) implies that the mixture's neutralization against these H1N1 viruses is dominated by the stem antibody, and hence there is no need to correct for the head antibody's neutralization.

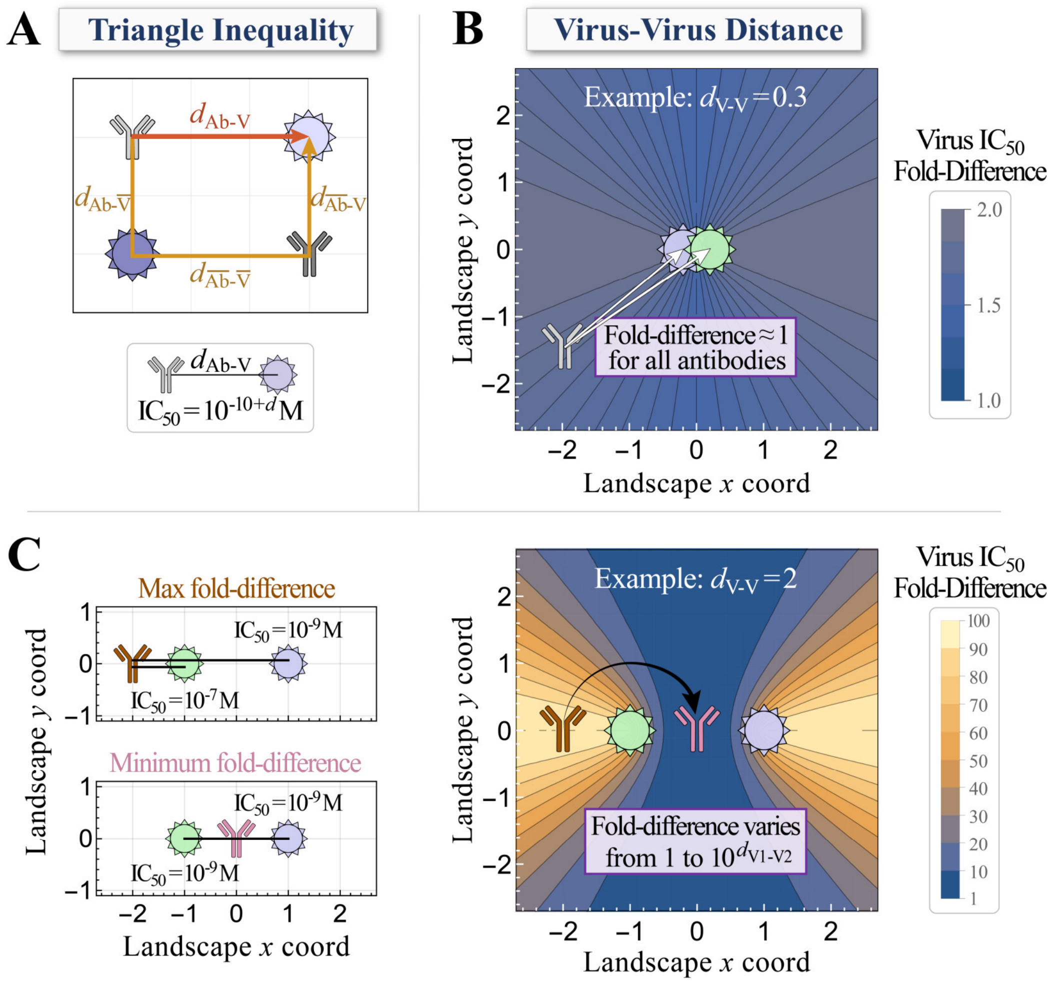

Supplementary Figure 6. Interpreting antibody-virus and virus-virus distance. (A) The triangle inequality is defined between an antibody (Ab, top-left) and virus (V, top-right) relative to any other antibody ($\overline{\text{Ab}}$, bottom-right) and virus ($\overline{\text{V}}$, bottom-left). (B) Virus-virus distance constrains the fold-difference in neutralization ($IC_{50,\text{Virus 1}}/IC_{50,\text{Virus 2}}$). When two viruses lie at nearly the same coordinate, all stem antibodies will neutralize them identically. (C) More generally, when two viruses are separated by a distance $d_{V\text{-}V}$, the fold-difference in neutralization for these two viruses can vary from the maximum value $10^{d_{V\text{-}V}}$ (brown antibody) to the minimum value of 1 when both viruses are equally neutralized (pink antibody).

Finally, recalling that antibodies and viruses are treated symmetrically in our framework, we note that antibody-antibody distance has an analogous interpretation, constraining how differently any virus can be neutralized by both antibodies. Two antibodies at the same location will exhibit identical neutralization profiles, whereas two antibodies a distance $d_{Ab\text{-}Ab}$ apart will neutralize any virus at most $10^{d_{Ab\text{-}Ab}}$-fold differently. We note that these are all idealized properties of the Neutralization Landscape that rely upon its underlying structure accurately reflecting the data.

The Tradeoff between Antibody Potency and Breadth

Using the Neutralization Landscape, we can computationally explore how potently any set of viruses can be neutralized by an optimal antibody (*i.e.*, an antibody with the smallest possible IC_{50} against every virus in the set). By construction, when viruses are spread out across the landscape, then no stem antibody can potently neutralize them all (Figure S1E). Using each point on the landscape to represent a potential antibody, we see that the resulting neutralization profiles of such an antibody are highly restricted. In particular, an optimal antibody that broadly neutralizes highly distinct viruses will necessarily be less potent than more-specific antibodies that only neutralize a subset of these viruses.

For example, in Figure 3 we explored how an antibody can inhibit all H3N2 vaccine strains from the 2004-05 to the 2018-19 season (A/Fujian/411/2002, A/California/07/2004, A/Wisconsin/67/2005, A/Brisbane/10/2007, A/Perth/16/2009, A/Victoria/361/2011, A/Texas/50/2012, A/Switzerland/9715293/2013, A/Hong Kong/4801/2014, and A/Singapore/INFIMH-160019/2016) and all H1N1 vaccine strains from this same period (A/New Caledonia/20/1999, A/Solomon Islands/03/2006, A/Brisbane/59/2007 [substituted with A/New York/08-1326/2008, see next paragraph], A/California/07/2009, and A/Michigan/45/2015). The optimal antibody that neutralizes all these distinct strains can only have an $IC_{50} \leq 30 \cdot 10^{-10}$ M against these viruses; in contrast, an optimal antibody against just the H1N1 strains can achieve an $IC_{50} \leq 3 \cdot 10^{-10}$ M (a 10-fold improvement) while an antibody targeting just the H3N2 vaccine strains can achieve an $IC_{50} \leq 4 \cdot 10^{-10}$ M.

We note that of these 15 vaccine strains, 14 were in our virus panel. The one missing strain, A/Brisbane/59/2007, was substituted with A/New York/08-1326/2008 whose HA sequence only differs by 2 amino acids. Given this sequence similarity, the two viruses should have similar neutralization profiles and hence have similar positions on the landscape. In general, the ability to substitute viruses based on their sequences greatly expands the utility of the Neutralization Landscape.

B. Modeling Combinations of Antibodies

The workflow to decompose polyclonal serum can be split into three parts. First, we use monoclonal antibody data to create the Neutralization Landscape for the HA stem, and we use its structure to remove the neutralization from antibodies that do not target the HA stem (discussed in Section A above). Second, we determine how multiple stem antibodies collectively neutralize a virus (the purview of this section). Third, we combine the two previous points to determine which combination of stem antibodies best describes a mixture's neutralization data (explained in section C).

Here, we tackle the second part, namely, to create a model that takes the IC_{50}s of individual antibodies against a virus and predicts the IC_{50} of their combination. As described below, we utilize measurements from our 2-antibody and 3-antibody mixtures to conclude that antibody combinations are well characterized by a competitive binding model, where all antibodies compete for the same region on HA.

Overview of Antibody Mixtures

We created 14 mixtures containing either two antibodies (at 1:1 stoichiometry) or three antibodies (at 1:1:1 stoichiometry) from our panel (Figure S7). We measured the neutralization of each combination against our virus panel as described previously (*10*). The IC_{50} of these antibody mixtures together with the IC_{50}s of the monoclonal antibodies are provided in the GitHub file "*(2) Neutralization Data for Monoclonal Antibodies and Mixtures.csv*".

Throughout this work, the mixture IC_{50}s represent the total concentration of IgGs at which 50% of the virus was neutralized. For example, Mixture #7 comprising Stem Ab 27 + Head Ab 1 neutralized H1N1 A/Weiss/1943 with an IC_{50} of $5.25\cdot10^{-9}$ M, so that each individual antibody was present at half this concentration at the point of 50% neutralization.

The following section describes the competitive binding model we developed to test how each antibody's individual neutralization could predict the mixture's combined neutralization. Since all antibodies on our panel bind to the same region on the HA stem (*32–34*), we assumed competitive binding where only one antibody can bind an HA monomer at a time due to steric exclusion (Figure 5A) (*28*). We note that IgG is a bulky molecule, and the size of each of its two Fabs (8 nm×5 nm×4 nm) is roughly comparable to an HA trimer (cylinder with length 13.5 nm and diameter 5.5 nm) (*28*). Thus, there is little room for two antibodies to bind to the stem [Figure S9 of Ref. (*35*)], especially considering that HA trimers are tightly packed on the surface of the influenza virus with a mean separation of ~14 nm (*28*).

Collective Neutralization of Stem Antibody Mixtures is Well Characterized by Competitive Binding

We begin by considering a two-antibody mixture with total IgG concentration c. This mixture is composed of a fraction f_1 of the first antibody and a fraction f_2=1-f_1 of the second antibody, so that the antibodies are at concentrations c_1=f_1c and c_2=f_2c. Let $IC_{50}^{(1)}$ be the concentration of the first antibody necessary for 50% inhibition (by itself) and $IC_{50}^{(2)}$ be the analogous value for the second antibody.

Figure 5A enumerates the possible states within each model, using a simplified representation for a binding site on viral HA (purple) where each antibody can bind at a single site. A virus is assumed to be neutralized if any antibodies are bound to it. Hence, neutralization is given by

$$\text{Neutralization}_{\text{Competitive}} = \frac{\frac{c_1}{\text{IC}_{50}^{(1)}} + \frac{c_2}{\text{IC}_{50}^{(2)}}}{1 + \frac{c_1}{\text{IC}_{50}^{(1)}} + \frac{c_2}{\text{IC}_{50}^{(2)}}}$$

in the competitive binding model.

For each model, the mixture IC_{50} is defined as the total IgG concentration c for which the neutralization formula equals 0.5, representing that a virion has a 50% chance of being neutralized. Hence

$$\text{IC}_{50,\text{Competitive}}^{\text{Mixture}} = \left(\sum_j \frac{f_j}{\text{IC}_{50}^{(j)}}\right)^{-1}$$

for the competitive binding model, and this formulation holds for mixtures with an arbitrary number of antibodies. With this equation, we predicted each mixture's IC_{50} from the IC_{50}s of the constituent antibodies. For our stem+stem mixtures (#8-11 in Figure S7, N=165 data points), over 98% of measurements had ≤4-fold error [Figure 5B]. This tight agreement implies that if there are any errors in the monoclonal antibody or antibody mixture data, the competitive binding model can readily flag those outliers so they can be remeasured.

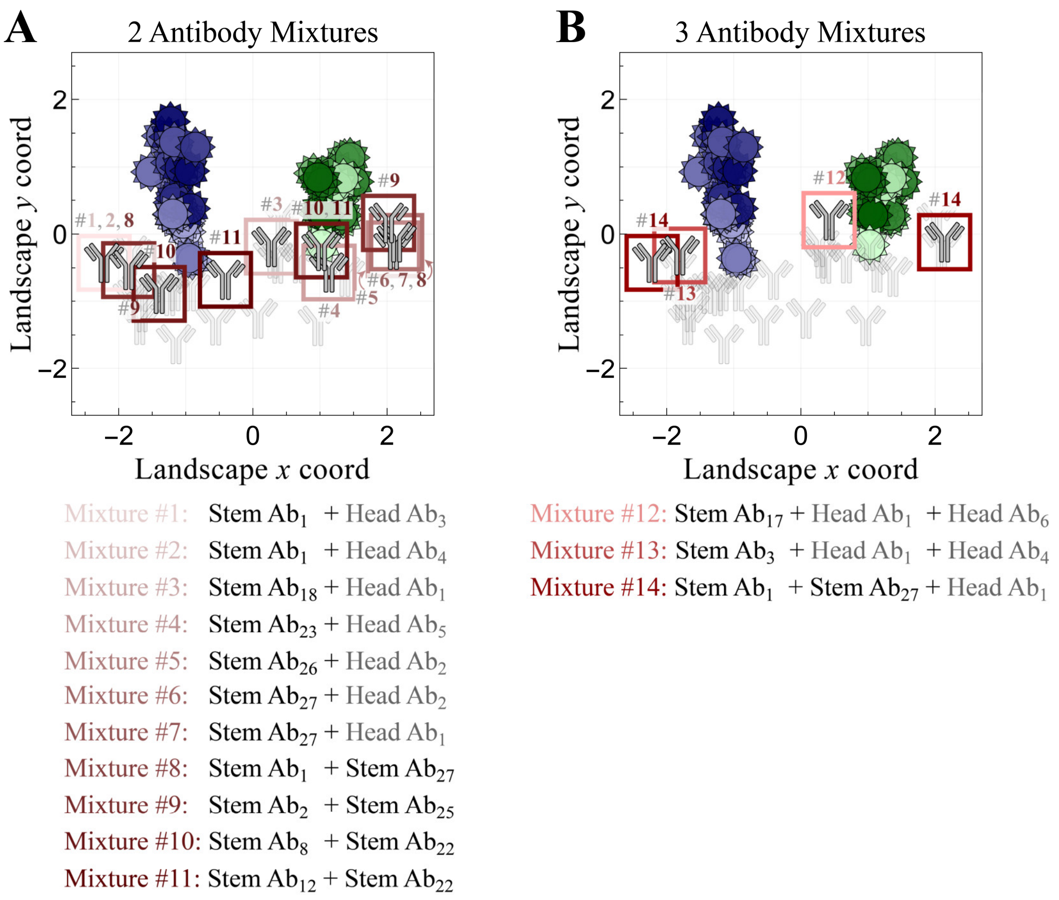

Supplementary Figure 7. Mixtures of two or three antibodies created to test the decomposition algorithm. We created 14 mixtures of head and stem antibodies containing (A) 2 antibodies or (B) 3 antibodies. The stem antibodies used in these mixtures are boxed and labeled with the mixtures containing them. See Table S1 for the full antibody names.

C. Decomposing Defined Antibody Mixtures

In this section, we combined the Neutralization Landscape (described in section A) with the mathematical framework to model multiple stem antibodies (section B). With this basis set of antibody behaviors, the total neutralization of an arbitrary antibody mixture against a virus panel can be decomposed into the minimal set of antibodies that can give rise to the observed measurements.

Why Polyclonal Mixtures need to be Represented using Multiple Points

To date, antigenic cartography has been based on the interactions between polyclonal ferret sera and different influenza strains, with each virus and each serum represented by a single point on a map (*2*, *36*). This representation of polyclonal sera raises an important question: if two sera are pooled together, could any single point accurately represent their mixture?

As a simple example, consider the scenario shown in Figure S8 where serum 1 potently neutralizes virus 1 while serum 2 strongly neutralizes virus 2, with virus 3 lying somewhere in between. Traditional antigenic cartography would suggest that the combination of serum 1+2 would lie between the two points representing the original sera, but any such point (and, in fact, any single point on the map) fails to capture the expected strong neutralization against viruses 1 and 2 and the weak neutralization against virus 3.

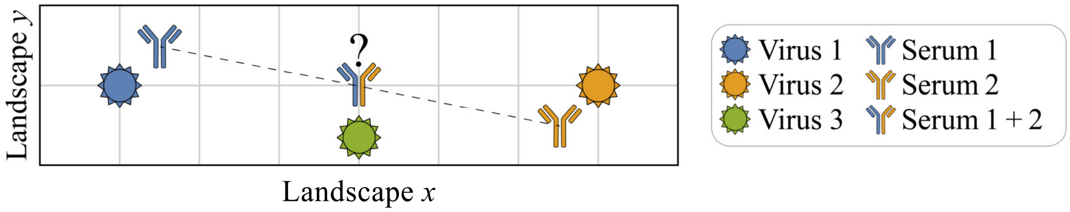

Supplementary Figure 8. Contradictions arise when representing polyclonal sera as single points. Serum 1 is individually potent against virus 1, while serum 2 is individually potent against virus 2. Thus, combining both sera (1:1 stoichiometry) should result in a serum potent against both viruses. If we use the approach from Smith *et al*. to represent this combined serum as a single point, then minimizing the error to viruses 1 and 2 would imply that it lies between the two viruses [blue/yellow marker in the center]. This incorrectly implies that the combined serum will weakly neutralize viruses 1 and 2, and moreover it suggests that the combination will neutralize virus 3 much more potently than should be possible by either serum 1 or 2 alone. Instead, the correct representation of the combined serum should be to use two markers (at the locations of serum 1 and 2).

Extending this logic to its extreme, if all serum samples analyzed in Smith *et al*. 2004 were combined, they should effectively inhibit all H3N2 strains analyzed, yet no single point on their map could be sufficiently close to the spread-out viruses to match this expected level of protection.

Instead, the cartography approach based on monoclonal antibody data suggests that mixtures of antibodies should be represented by multiple points. For example, the mixture of serum 1+2 in Figure S8 should be represented by two markers at the locations of serum 1 and serum 2. The more polyclonal a mixture is, the more potential error may be introduced when representing it by a single point. The following sections examine the consequences of this multi-point representation for polyclonal mixtures.

Defining Decomposition Error

We first define the error metric used to quantify how our predicted antibodies compare to the true mixture behavior. We compute the mean fold-error between the neutralization of our predicted stem antibodies and the mixture's measured neutralization,

$$\langle \text{decomposition error} \rangle = 1/N \sum \text{fold-error}[IC_{50}^{\text{predicted}}, IC_{50}^{\text{measured}}].$$

Note that $IC_{50}^{\text{measured}}$ quantifies the neutralization from just the stem antibodies within each mixture. This is experimentally accessible, since for each of our head+stem mixtures, we also created the stem mixture without the head component (Figure S7). More precisely, for mixtures #1-7 and #12-13 we know how the single stem antibody behaves from our monoclonal antibody data, while for mixture #14 we know how stem antibodies 1 + 27 behave from mixture #8. (The remaining mixtures #8-11 do not include any head antibodies.) Therefore, in each case, we can compare the predictions of the stem antibodies within a mixture against the measurements of a mixture containing only those stem antibodies.

$IC_{50}^{\text{predicted}}$ incorporates all predicted stem antibodies and their stoichiometry via the competitive binding model (see the formula for $IC_{50,\text{Competitive}}^{\text{Mixture}}$ in Section B). Following Smith *et al.*, whenever a measurement was given as a bound and the prediction obeyed this bound (*e.g.*, $IC_{50}^{\text{measured}} > 10^{-7}$ M and $IC_{50}^{\text{predicted}} = 10^{-6}$ M), we excluded measurement-prediction pairs from the summation above to prevent artificially *deflating* the error. However, when the measurement was given as a bound and the prediction did not satisfy this bound (*e.g.*, $IC_{50}^{\text{measured}} > 10^{-7}$ M and $IC_{50}^{\text{predicted}} = 10^{-8}$ M), we included this pair in the error calculation with $IC_{50}^{\text{measured}}$ equal to its bound.

Choosing the Subset of Viruses for Decomposition

While we measured the neutralization of each antibody mixture against our entire 51 virus panel, performing decompositions using this full panel may provide sub-optimal results since (1) some viruses may be less certain on the landscape and hurt the decompositions and (2) some regions on the map may be overrepresented and hence be overfit by the full virus panel. The most pressing concern is that a mixture containing multiple antibodies would be decomposed as containing a single antibody.

Following the technique employed with antibody fingerprinting (*5*), we determined which subset of viruses could best identify the two distinct antibodies within our stem+stem mixtures (Figure S7, Mixtures #8-11). We calculated the error ratio= $\frac{\text{error from "ground truth" decomposition}}{\text{error from a 1}-\text{antibody decomposition}}$, where the numerator equals the decomposition error using the actual coordinates and stoichiometries of the 2-antibody mixture, while the denominator represents the error from incorrectly decomposing this mixture as a single antibody. A lower error ratio signifies that the mixture can be better described as a mixture containing more than 1 antibody, and as described in the following section, an error ratio$<$0.8 ensures that the mixture decomposition will contain at least 2 antibodies (since the optimal 2-antibody decomposition will have at most the decomposition error of the "ground truth" decomposition).

Although singly removing each virus had a small effect (always between 0.96 and 1.0), removing multiple viruses decreased the mean error ratio across our stem+stem mixtures. Thus, we ordered the viruses from the largest-to-smallest drop in error ratio, and we removed the top *n* viruses. The mean error

ratio across our stem+stem mixtures decreased with each additional virus removed until $n = 20$ where it plateaued at 0.8, representing a virus panel that is substantially better at detecting multiple stem antibodies. Therefore, in our decompositions of all monoclonal stem antibodies and stem mixtures (Figure S10), we only use neutralization measurements from the following 31 viruses:

- H1N1: A/WSN/1933, A/Puerto Rico/8/1934, A/Weiss/1943, A/Malaysia/1954, A/Kiev/1/1957, A/Beijing/262/1995, A/New Caledonia/20/1999, A/Solomon Islands/03/2006, A/New York/08-1326/2008, A/California/07/2009, A/Boston/YGA-01050/2012, A/Idaho/07/2018
- H3N2: A/Aichi/2/1968, A/Philippines/2/1982, A/Colorado/2/1986, A/Sichuan/2/1987, A/Beijing/353/1989, A/Johannesburg/33/1994, A/Brisbane/8/1996, A/Sydney/5/1997, A/Moscow/10/1999, A/Fujian/411/2002, A/California/07/2004, A/Perth/16/2009, A/Indiana/10/2011, A/Victoria/361/2011, A/Texas/50/2012, A/Switzerland/9715293/2013, A/Hong Kong/4801/2014, A/Singapore/INFIMH-160019/2016, A/Perth/1008/2019

With this reduced virus panel, we correctly identified the multiple stem antibodies in all stem+stem mixtures as well as the head+stem+stem mixture.

Overview of the Decomposition Algorithm

Serum decomposition proceeds by characterizing a mixture using an increasing number of antibodies, halting once the addition of another antibody no longer markedly decreases the decomposition error.

1. Remove any head antibody neutralization (described in SI Section A).
2. Start by describing a mixture with n=1 antibody, and then consider n=2, 3… antibodies (as shown in Figure S9A,B).
3. Starting from n antibodies, we accept the best decomposition with n+1 antibodies if the ⟨decomposition error⟩ decreases by at least 20%. This ensures that adding another antibody markedly reduces the error.

When the decomposition with n+1 antibodies decreases ⟨decomposition error⟩ by less than 20%, the search terminates and returns the best decomposition containing n antibodies.

At each step, we consider all possible positions for the n antibodies and (for $n \geq 2$) all possible stoichiometries, but we force each antibody to comprise ≥10% of the mixture to ensure that its neutralization can be clearly detected (since an antibody comprising ~1% of a mixture will negligibly affects the mixture's neutralization). For example, for a mixture comprising 1 head+1 stem antibody, we consider everything from a 10%/90% composition to a 90%/10% composition.

Figure S9 shows two example decompositions for mixture #1 [top row] and mixture #14 [bottom row]. In the top row, we first remove the head neutralization signal. We then begin with a decomposition using n= 1 antibody. The transition from n=1→2 antibodies decreases the input error by only 5%, correctly indicating that the mixture contains 1 stem antibody (shown in gray; the second red antibody is off the landscape on the top-left). Note that in this figure only, we show $\text{Error}_{\text{Input}}$, that is, the error describing how well a decomposition with n antibodies reproduces the input mixture neutralization. When we show the error in all other figures, it is relative to the stem antibodies within the mixture (e.g., for mixture #1, we would compare the neutralization predicted by each decomposition against the neutralization from stem antibody #1 which is in that mixture). However, to determine the number of stem antibodies within each mixture, we are only allowed to use the input signal.

For mixture #14 shown in the bottom row of Figure S9, we begin with $n=1$ antibody. The transition from $n=1\rightarrow2$ decreases input error by 45% and is accepted, but $n=2\rightarrow3$ does not decrease the input error further and is rejected (the third red antibody is off the landscape on the bottom-left). Thus, the mixture is correctly predicted to have 2 stem antibodies, although the positions of these antibodies somewhat deviates from the ground truth. The fact that both the red and gray antibodies have nearly identical neutralization profiles (as seen by the small decomposition error) demonstrates that this set of neutralization measurements can arise from multiple sets of antibodies.

We note that while we have only quantified the optimal decomposition, we can also quantify how good the "next best" alternative is (containing either a different number of stem antibodies or a different position/stoichiometry for these antibodies) by comparing their ⟨decomposition error⟩. Thus, we can clarify when the transition between n and $n+1$ antibodies is just shy of the 20% threshold, or if different configurations of n antibodies have nearly identical error, thereby quantifying the degeneracy of the antibody response.

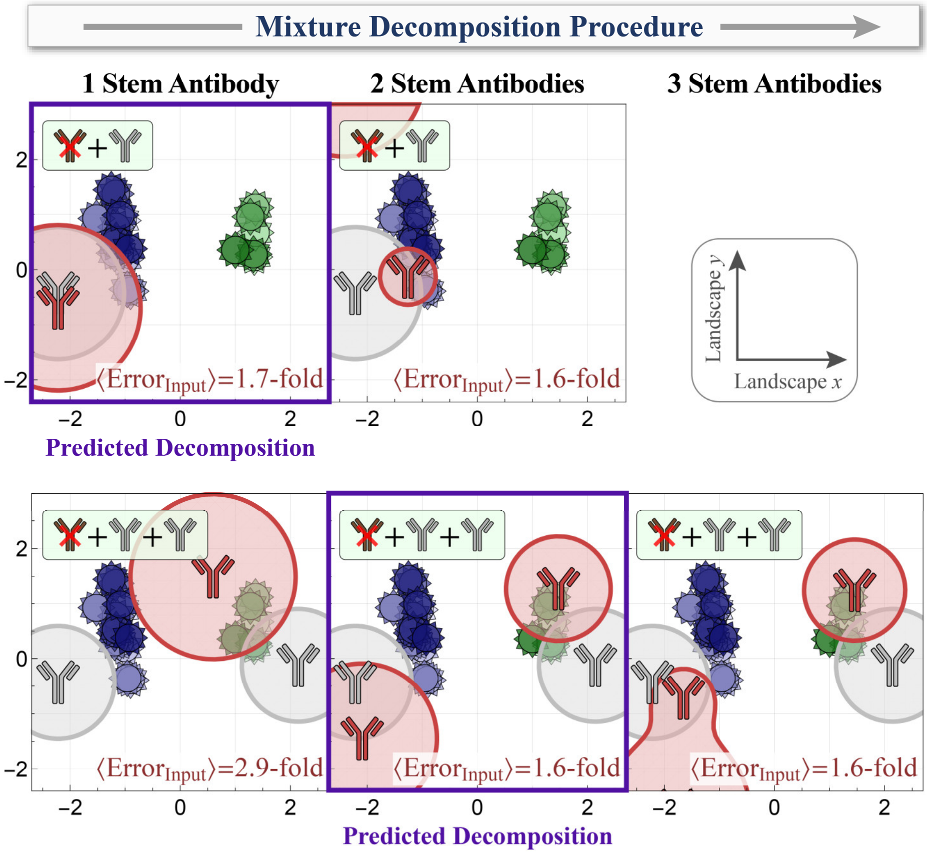

Supplementary Figure 9. Decomposition algorithm to detect the number of stem antibodies in a mixture. We decompose the collective neutralization of a mixture against our virus panel using an increasing number of antibodies, stopping when the error will not decrease by at least 20% with the addition of the next antibody. Examples are shown for (A) mixture #1 and (B) mixture #14. Decomposition proceeds by analyzing 1, 2, 3... antibodies, halting once the error decreases by less than 20%. The predicted decomposition is boxed in purple. On each 2D landscape, the stem antibodies in the mixture are shown in gray while the antibodies predicted by decomposition are shown in red. The circles around each antibody show the region of virus neutralization when the mixture has a total concentration of $10^{-8.5}$ M.

Results from Decomposing Antibody Mixtures

Figure S10 shows the decompositions for our 14 antibody mixtures. On each plot, we show the location of the actual (gray) and inferred (red) antibodies that collectively give rise to the mixture's neutralization profile. Panel D shows the number of inferred antibodies and their stoichiometry. These decomposition results are also available in the GitHub file "*(3) Decomposition Results.csv*", where each antibody in the decomposition is classified using the format {(map coordinates), fractional composition of the stem response}.

For example, as shown for mixture #11 in Figure S10C, we predict that there are two stem antibodies with 70%/30% stoichiometry, whereas the true mixture has an equal 50%/50% composition (since this head+stem+stem mixture has equal amounts of each antibody, so after removing the head antibody the two stem antibodies comprise 50%/50% of the mixture). The radius of the circular regions surrounding each antibody corresponds to the region of ≥50% neutralization when the total IgG concentration of stem antibodies in the mixture equals $10^{-8.5}$ M. Thus, the gray circles surrounding the actual antibodies have a radius of $1.5+\log_{10}(½)=1.2$, where 1.5 corresponds to the total mixture concentration of $10^{-8.5}$ M while $\log_{10}(½)$ accounts for the fact that each antibody only comprises half the mixture. The radius of the red circles around the predicted antibodies will be $1.5+\log_{10}(7/10)=1.3$ and $1.5+\log_{10}(3/10)=1.0$ for the red antibody on the left and right, respectively. In addition, the two red circles are elongated and merged because the two antibodies can both neutralize the virus, as seen by the solid versus dashed lines in Figure 5C (with the gray circles similarly elongated). In each panel, we only show the subset of viruses (described in the preceding section) that each mixture was measured against — in particular, mixtures #4, 5, 10, and 11 were measured against slightly fewer viruses because of limited reagents.

In only a single case (mixture #3), we predicted the wrong number of stem antibodies, inferring two antibodies instead of one stem antibody. Moreover, if we decompose the neutralization of the 27 monoclonal antibodies in our panel, 23/27 are correctly decomposed as containing a single stem antibody while the other 4 are overfit as having two stem antibodies. Such cases happen when the 2D map representation for an antibody has large error, or when a different antibody configuration happens to more closely match the antibody's neutralization profile. In the majority of cases, decomposition determines the correct number of stem antibodies, albeit with potentially large deviations in the stoichiometry. Notably, in *all* cases the resulting decompositions have ≲6-fold error compared to the true mixture. Given that the collective neutralization measurements must determine the number of antibodies, their locations on the map, and their fractional compositions, this low error highlights the wealth of information encoded by the virus coordinates on a Neutralization Map.

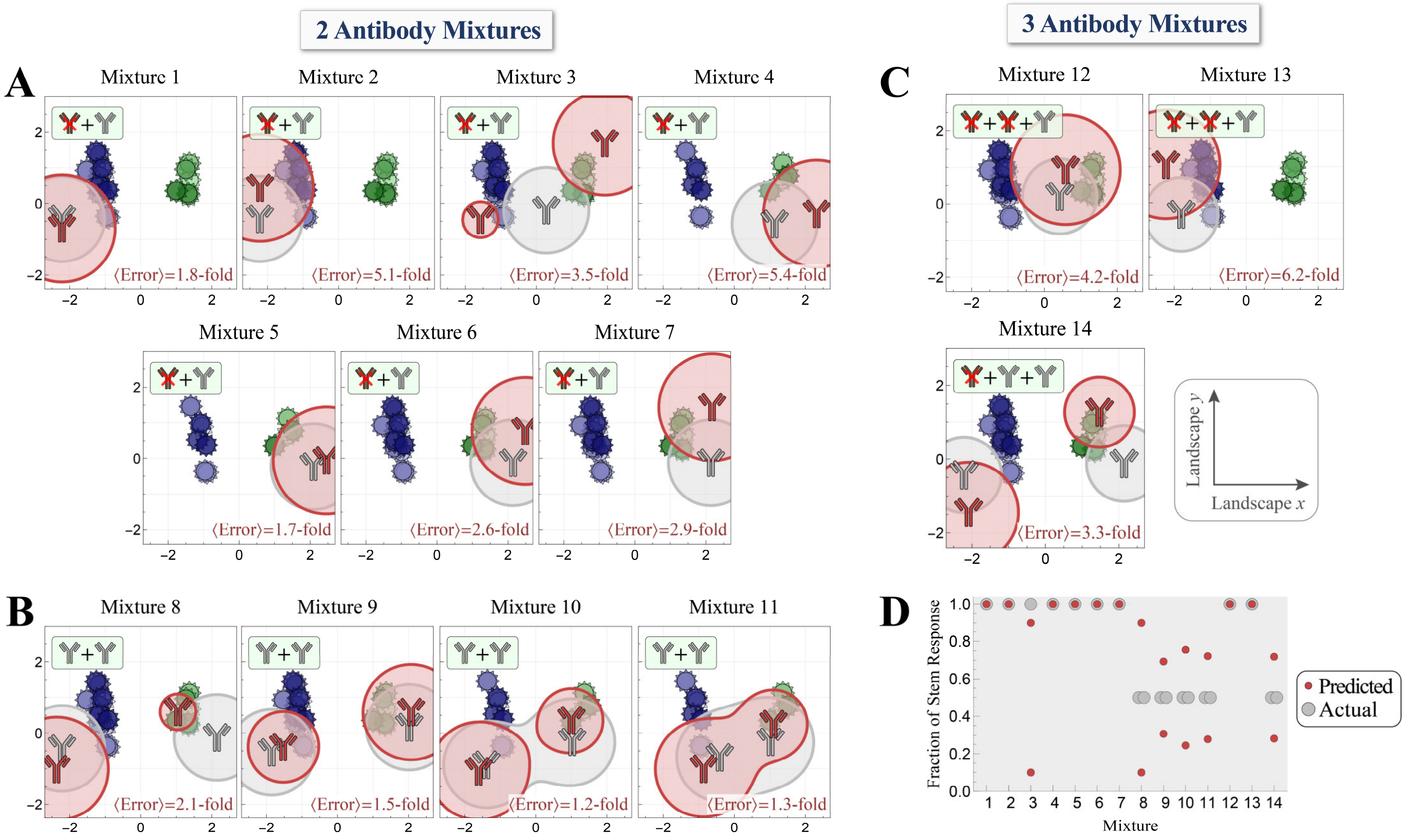

Supplementary Figure 10. Decomposing of all antibody mixtures. For each mixture, we computationally remove the neutralization from any head antibodies and predict the optimal number, stoichiometry, and neutralization profiles of the stem antibodies within. Decomposition was performed without any knowledge of the antibodies within the mixture. Results for (A) head+stem, (B) stem+stem, or (C) head+head+stem or head+stem+stem mixtures. (D) The resulting stoichiometry of the predicted vs actual antibodies, shown as the fraction of the stem antibody response (since the neutralization from head antibodies was removed).

References

1. A. Singer, A remark on global positioning from local distances. *Proc. Natl. Acad. Sci.* **105**, 9507–9511 (2008).
2. D. J. Smith, A. S. Lapedes, J. C. de Jong, T. M. Bestebroer, G. F. Rimmelzwaan, A. D. M. E. Osterhaus, R. A. M. Fouchier, Mapping the antigenic and genetic evolution of influenza virus. *Science*. **305**, 371–376 (2004).
3. J. Foote, H. N. Eisen, Kinetic and affinity limits on antibodies produced during immune responses. *Proc. Natl. Acad. Sci. U. S. A.* **92**, 1254–6 (1995).
4. S. F. Andrews, Y. Huang, K. Kaur, L. I. Popova, I. Y. Ho, N. T. Pauli, C. J. Henry Dunand, W. M. Taylor, S. Lim, M. Huang, X. Qu, J.-H. Lee, M. Salgado-Ferrer, F. Krammer, P. Palese, J. Wrammert, R. Ahmed, P. C. Wilson, Immune history profoundly affects broadly protective b cell responses to influenza. *Sci. Transl. Med.* **7**, 316ra192 (2015).
5. I. S. Georgiev, N. A. Doria-Rose, T. Zhou, Y. Do Kwon, R. P. Staupe, S. Moquin, G. Y. Chuang, M. K. Louder, S. D. Schmidt, H. R. Altae-Tran, R. T. Bailer, K. McKee, M. Nason, S. O'Dell, G. Ofek, M. Pancera, S. Srivatsan, L. Shapiro, M. Connors, S. A. Migueles, L. Morris, Y. Nishimura, M. A. Martin, J. R. Mascola, P. D. Kwong, Delineating antibody recognition in polyclonal sera from patterns of hiv-1 isolate neutralization. *Science*. **340**, 751–756 (2013).
6. K. Wagh, T. Bhattacharya, C. Williamson, A. Robles, M. Bayne, J. Garrity, M. Rist, C. Rademeyer, H. Yoon, A. Lapedes, H. Gao, K. Greene, M. K. Louder, R. Kong, S. A. Karim, D. R. Burton, D. H. Barouch, M. C. Nussenzweig, J. R. Mascola, L. Morris, D. C. Montefiori, B. Korber, M. S. Seaman, Optimal combinations of broadly neutralizing antibodies for prevention and treatment of hiv-1 clade c infection. *PLOS Pathog.* **12**, e1005520 (2016).
7. J. E. Crowe, Is it possible to develop a "universal" influenza virus vaccine? *Cold Spring Harb. Perspect. Biol.* **10**, a029496 (2018).
8. S. Omar Ali, T. Takas, A. Nyborg, K. Shoemaker, N. L. Kallewaard, R. Chiong, F. Dubovsky, R. M. Mallory, Evaluation of medi8852, an anti-influenza a monoclonal antibody, in treating acute uncomplicated influenza. *Antimicrob. Agents Chemother.* **62** (2018) (available at https://doi.org/10.1128/AAC.00694-18).
9. D. J. DiLillo, G. S. Tan, P. Palese, J. V Ravetch, Broadly neutralizing hemagglutinin stalk–specific antibodies require fcγr interactions for protection against influenza virus in vivo. *Nat. Med. 2014 202*. **20**, 143–151 (2014).
10. A. Creanga, R. A. Gillespie, B. E. Fisher, S. F. Andrews, J. Lederhofer, C. Yap, L. Hatch, T. Stephens, Y. Tsybovsky, M. C. Crank, J. E. Ledgerwood, A. B. McDermott, J. R. Mascola, B. S. Graham, M. Kanekiyo, A comprehensive influenza reporter virus panel for high-throughput deep profiling of neutralizing antibodies. *Nat. Commun.* **12**, 1722 (2021).

11. A. D. DeZure, E. E. Coates, Z. Hu, G. V. Yamshchikov, K. L. Zephir, M. E. Enama, S. H. Plummer, I. J. Gordon, F. Kaltovich, S. Andrews, A. McDermott, M. C. Crank, R. A. Koup, R. M. Schwartz, R. T. Bailer, X. Sun, J. R. Mascola, T. M. Tumpey, B. S. Graham, J. E. Ledgerwood, An avian influenza h7 dna priming vaccine is safe and immunogenic in a randomized phase i clinical trial. *NPJ vaccines*. **2** (2017), doi:10.1038/S41541-017-0016-6.
12. S. F. Andrews, M. Gordon Joyce, M. J. Chambers, R. A. Gillespie, M. Kanekiyo, K. Leung, E. S. Yang, Y. Tsybovsky, A. K. Wheatley, M. C. Crank, J. C. Boyington, M. S. Prabhakaran, S. R. Narpala, X. Chen, R. T. Bailer, G. Chen, E. Coates, P. D. Kwong, R. A. Koup, J. R. Mascola, B. S. Graham, J. E. Ledgerwood, A. B. McDermott, Preferential induction of cross-group influenza a hemagglutinin stem–specific memory b cells after h7n9 immunization in humans. *Sci. Immunol.* **2** (2017) (available at https://doi.org/10.1126/sciimmunol.aan2676).
13. J. E. Ledgerwood, K. Zephir, Z. Hu, C. J. Wei, L. Chang, M. E. Enama, C. S. Hendel, S. Sitar, R. T. Bailer, R. A. Koup, J. R. Mascola, G. J. Nabel, B. S. Graham, Prime-boost interval matters: a randomized phase 1 study to identify the minimum interval necessary to observe the h5 dna influenza vaccine priming effect. *J. Infect. Dis.* **208**, 418–422 (2013).
14. N. C. Wu, S. F. Andrews, J. E. Raab, S. O'Connell, C. A. Schramm, X. Ding, M. J. Chambers, K. Leung, L. Wang, Y. Zhang, J. R. Mascola, D. C. Douek, J. E. Ledgerwood, A. B. McDermott, I. A. Wilson, Convergent evolution in breadth of two v h 6-1-encoded influenza antibody clonotypes from a single donor. *Cell Host Microbe*. **28**, 434-444.e4 (2020).
15. M. Throsby, E. van den Brink, M. Jongeneelen, L. L. M. Poon, P. Alard, L. Cornelissen, A. Bakker, F. Cox, E. van Deventer, Y. Guan, J. Cinatl, J. ter Meulen, I. Lasters, R. Carsetti, M. Peiris, J. de Kruif, J. Goudsmit, Heterosubtypic neutralizing monoclonal antibodies cross-protective against h5n1 and h1n1 recovered from human igm+ memory b cells. *PLoS One*. **3** (2008), doi:10.1371/JOURNAL.PONE.0003942.
16. D. C. Ekiert, R. H. E. Friesen, G. Bhabha, T. Kwaks, M. Jongeneelen, W. Yu, C. Ophorst, F. Cox, H. J. W. M. Korse, B. Brandenburg, R. Vogels, J. P. J. Brakenhoff, R. Kompier, M. H. Koldijk, L. A. H. M. Cornelissen, L. L. M. Poon, M. Peiris, W. Koudstaal, I. A. Wilson, J. Goudsmit, A highly conserved neutralizing epitope on group 2 influenza a viruses. *Science*. **333**, 843–850 (2011).
17. Y. Wu, M. Cho, D. Shore, M. Song, J. Choi, T. Jiang, Y.-Q. Deng, M. Bourgeois, L. Almli, H. Yang, L.-M. Chen, Y. Shi, J. Qi, A. Li, K. S. Yi, M. Chang, J. S. Bae, H. Lee, J. Shin, J. Stevens, S. Hong, C.-F. Qin, G. F. Gao, S. J. Chang, R. O. Donis, A potent broad-spectrum protective human monoclonal antibody crosslinking two haemagglutinin monomers of influenza a virus. *Nat. Commun.* **6**, 7708 (2015).
18. D. Corti, J. Voss, S. J. Gamblin, G. Codoni, A. Macagno, D. Jarrossay, S. G. Vachieri, D. Pinna, A. Minola, F. Vanzetta, C. Silacci, B. M. Fernandez-Rodriguez, G. Agatic, S. Bianchi, I. Giacchetto-Sasselli, L. Calder, F. Sallusto, P. Collins, L. F. Haire, N. Temperton, J. P. M. Langedijk, J. J. Skehel, A. Lanzavecchia, A neutralizing antibody selected from plasma cells that binds to group 1 and group 2 influenza a hemagglutinins. *Science*. **333**, 850–856 (2011).
19. N. L. Kallewaard, D. Corti, P. J. Collins, U. Neu, J. M. McAuliffe, E. Benjamin, L. Wachter-Rosati, F. J. Palmer-Hill, A. Q. Yuan, P. A. Walker, M. K. Vorlaender, S. Bianchi, B. Guarino, A. De Marco, F. Vanzetta, G. Agatic, M. Foglierini, D. Pinna, B. Fernandez-Rodriguez, A. Fruehwirth, C. Silacci, R. W. Ogrodowicz, S. R. Martin, F. Sallusto, J. A. Suzich, A. Lanzavecchia, Q. Zhu, S. J. Gamblin, J. J. Skehel, Structure and function analysis of an antibody recognizing all influenza a subtypes. *Cell*. **166**, 596–608 (2016).
20. T. Einav, Y. Khoo, A. Singer, Quantitatively visualizing bipartite datasets. *arXiv* (2022), doi:10.48550/arxiv.2207.12658.
21. M. B. Doud, J. D. Bloom, Accurate measurement of the effects of all amino-acid mutations on influenza hemagglutinin. *Viruses*. **8** (2016), doi:10.3390/V8060155.
22. E. Kirkpatrick, X. Qiu, P. C. Wilson, J. Bahl, F. Krammer, The influenza virus hemagglutinin head evolves faster than the stalk domain. *Sci. Rep.* **8** (2018), doi:10.1038/S41598-018-28706-1.
23. E. K. Roubidoux, J. M. Carreño, M. McMahon, K. Jiang, H. van Bakel, F. Krammer, P. Wilson, Mutations in the hemagglutinin stalk domain do not permit escape from a protective, stalk-based vaccine-induced immune response in the mouse model. *MBio*. **12**, 1–14 (2021).
24. T. Bedford, M. A. Suchard, P. Lemey, G. Dudas, V. Gregory, A. J. Hay, J. W. McCauley, C. A. Russell, D. J. Smith, A. Rambaut, Integrating influenza antigenic dynamics with molecular evolution. *Elife*. **3** (2014), doi:10.7554/eLife.01914.
25. S. R. Christensen, E. T. Martin, J. G. Petrie, A. S. Monto, S. E. Hensley, *bioRxiv*, in press, doi:10.1101/2022.01.03.474869.
26. J. M. Lee, J. Huddleston, M. B. Doud, K. A. Hooper, N. C. Wu, T. Bedford, J. D. Bloom, Deep mutational scanning of hemagglutinin helps predict evolutionary fates of human h3n2 influenza variants. *Proc. Natl. Acad. Sci.* **115**, E8276–E8285 (2018).
27. N. C. Wu, A. J. Thompson, J. M. Lee, W. Su, B. M. Arlian, J. Xie, R. A. Lerner, H. L. Yen, J. D. Bloom, I. A. Wilson, Different genetic barriers for resistance to ha stem antibodies in influenza h3 and h1 viruses. *Science*. **368**, 1335–1340 (2020).
28. T. Einav, L. E. Gentles, J. D. Bloom, SnapShot: influenza by the numbers. *Cell*. **182**, 532-532.e1 (2020).
29. S. F. Andrews, A. B. McDermott, Shaping a universally broad antibody response to influenza amidst a variable immunoglobulin landscape. *Curr. Opin. Immunol.* **53**, 96–101 (2018).
30. A. M. Phillips, K. R. Lawrence, A. Moulana, T. Dupic, J. Chang, M. S. Johnson, I. Cvijovic, T. Mora, A. M. Walczak, M. M. Desai, Binding affinity landscapes constrain the evolution of broadly neutralizing anti-influenza antibodies. *Elife*. **10** (2021), doi:10.7554/ELIFE.71393.
31. G. Nakamura, N. Chai, S. Park, N. Chiang, Z. Lin, H. Chiu, R. Fong, D. Yan, J. Kim, J. Zhang, W. P. Lee, A. Estevez, M. Coons, M. Xu, P. Lupardus, M. Balazs, L. R. Swem, An in vivo human-plasmablast enrichment technique allows rapid identification of therapeutic influenza a antibodies. *Cell Host Microbe*. **14**, 93–103 (2013).
32. M. G. Joyce, A. K. Wheatley, P. V. Thomas, G. Y. Chuang, C. Soto, R. T. Bailer, A. Druz, I. S. Georgiev, R. A. Gillespie, M. Kanekiyo, W. P. Kong, K. Leung, S. N. Narpala, M. S. Prabhakaran, E. S. Yang, B. Zhang, Y. Zhang, M. Asokan, J. C. Boyington, T. Bylund, S. Darko, C. R. Lees, A. Ransier, C. H. Shen, L. Wang, J. R. Whittle, X. Wu, H. M. Yassine, C. Santos, Y. Matsuoka, Y. Tsybovsky, U. Baxa, J. C. Mullikin, K. Subbarao, D. C. Douek, B. S. Graham, R. A. Koup, J. E. Ledgerwood, M. Roederer, L. Shapiro, P. D. Kwong, J. R. Mascola, A. B. McDermott, Vaccine-induced antibodies that neutralize group 1 and group 2 influenza a viruses. *Cell*. **166**, 609–623 (2016).
33. S. F. Andrews, B. S. Graham, J. R. Mascola, A. B. McDermott, Is it possible to develop a "universal" influenza virus vaccine? *Cold Spring Harb. Perspect. Biol.* **10**, a029413 (2018).
34. N. C. Wu, I. A. Wilson, Influenza hemagglutinin structures and antibody recognition. *Cold Spring Harb. Perspect. Med.* **10**, a038778 (2020).
35. A. K. Harris, J. R. Meyerson, Y. Matsuoka, O. Kuybeda, A. Moran, D. Bliss, S. R. Das, J. W. Yewdell, G. Sapiro, K. Subbarao, S. Subramaniam, Structure and accessibility of ha trimers on intact 2009 h1n1 pandemic influenza virus to stem region-specific neutralizing antibodies. *Proc. Natl. Acad. Sci.* **110**, 4592–4597 (2013).
36. J. M. Fonville, S. H. Wilks, S. L. James, A. Fox, M. Ventresca, M. Aban, L. Xue, T. C. Jones, N. M. H. Le, Q. T. Pham, N. D. Tran, Y. Wong, A. Mosterin, L. C. Katzelnick, D. Labonte, T. T. Le, G. van der Net, E. Skepner, C. A. Russell, T. D. Kaplan, G. F. Rimmelzwaan, N. Masurel, J. C. de Jong, A. Palache, W. E. P. Beyer, Q. M. Le, T. H. Nguyen, H. F. L. Wertheim, A. C. Hurt, A. D. M. E. Osterhaus, I. G. Barr, R. A. M. Fouchier, P. W. Horby, D. J. Smith, Antibody landscapes after influenza virus infection or vaccination. *Science*. **346**, 996–10001 (2014).
