# Supplementary material for: Harnessing low dimensionality to visualize the antibody–virus landscape for influenza
Source: Nat Comput Sci. 2022 Dec 30;3(2):164–73. doi: 10.1038/s43588-022-00375-1 (PMC10766546; doi:10.1038/s43588-022-00375-1)
Supplement: Supplementary file 2 — Reporting Summary [file 43588_2022_375_MOESM2_ESM.pdf]

nature research

Corresponding author(s): Tal Einav

Last updated by author(s): Dec 21, 2022

Reporting Summary

Nature Research wishes to improve the reproducibility of the work that we publish. This form provides structure for consistency and transparency in reporting. For further information on Nature Research policies, see our Editorial Policies and the Editorial Policy Checklist.

Statistics

For all statistical analyses, confirm that the following items are present in the figure legend, table legend, main text, or Methods section.

n/a	Confirmed	
☐	☒	The exact sample size (n) for each experimental group/condition, given as a discrete number and unit of measurement
☐	☒	A statement on whether measurements were taken from distinct samples or whether the same sample was measured repeatedly
☒	☐	The statistical test(s) used AND whether they are one- or two-sided<br>*Only common tests should be described solely by name; describe more complex techniques in the Methods section.*
☐	☒	A description of all covariates tested
☐	☒	A description of any assumptions or corrections, such as tests of normality and adjustment for multiple comparisons
☐	☒	A full description of the statistical parameters including central tendency (e.g. means) or other basic estimates (e.g. regression coefficient) AND variation (e.g. standard deviation) or associated estimates of uncertainty (e.g. confidence intervals)
☒	☐	For null hypothesis testing, the test statistic (e.g. F, t, r) with confidence intervals, effect sizes, degrees of freedom and P value noted<br>*Give P values as exact values whenever suitable.*
☒	☐	For Bayesian analysis, information on the choice of priors and Markov chain Monte Carlo settings
☐	☒	For hierarchical and complex designs, identification of the appropriate level for tests and full reporting of outcomes
☒	☐	Estimates of effect sizes (e.g. Cohen's d, Pearson's r), indicating how they were calculated

Our web collection on statistics for biologists contains articles on many of the points above.

Software and code

Policy information about availability of computer code

Data collection	Plate image acquisition: Celigo v4.
Data analysis	Neutralization data was analyzed using GraphPad Prism 8. All subsequent data analysis was performed using Mathematica (Version 13.0.0). The supplementary notebook contains the full analysis and reproduces all plots from this work.

For manuscripts utilizing custom algorithms or software that are central to the research but not yet described in published literature, software must be made available to editors and reviewers. We strongly encourage code deposition in a community repository (e.g. GitHub). See the Nature Research guidelines for submitting code & software for further information.

Data

Policy information about availability of data

All manuscripts must include a data availability statement. This statement should provide the following information, where applicable:

- Accession codes, unique identifiers, or web links for publicly available datasets
- A list of figures that have associated raw data
- A description of any restrictions on data availability

The antibody-virus neutralization dataset used in this work combined existing measurements from Creanga et al. [Reference #16] for (17 antibodies)×(49 viruses) with new measurements carried out in this work. The resulting dataset is provided in the Source Data, together with data used to create all main text and supplemental figures.

Field-specific reporting

Please select the one below that is the best fit for your research. If you are not sure, read the appropriate sections before making your selection.

☒ Life sciences ☐ Behavioural & social sciences ☐ Ecological, evolutionary & environmental sciences

For a reference copy of the document with all sections, see nature.com/documents/nr-reporting-summary-flat.pdf

Life sciences study design

All studies must disclose on these points even when the disclosure is negative.

Sample size	This work required few choices for sample size. We created the Neutralization Landscape using as large a panel of monoclonal antibodies (n=27) and viruses (n=51) as possible. We similarly created multiple antibody mixtures (n=14), choosing diverse sets of antibodies that would be positioned at different locations on the landscape.
Data exclusions	No data was excluded from our analysis.
Replication	We validated the antibody-virus landscapes using different subsets of our dataset (Figure 2). We validated the decomposition of antibody mixtures using the 27 monoclonal antibodies in our panel as well as 14 mixtures of these antibodies (Figures 4 and 5).
Randomization	For leave-one-out analysis (Figure 2, bottom row of Figure S3), we used an antibody's neutralization against 6 viruses to predict its neutralization against the other 45 viruses in our panel. As described in SI Section "Extrapolating the Behavior of New Antibodies," selection of these six viruses was partly random: we randomly chose 3 H1N1 viruses and 3 H3N2 viruses to triangulate an antibody, but biased the selection towards viruses that were spread out along their y-coordinates to ensure that each selection provided complementary information. In all of our other analysis, we used the entire antibody-virus dataset and hence had no need for randomization
Blinding	For the computational analysis, blinding for the leave-one-out or leave-some-out analyses was done by randomly drawing from available samples. Experimenters were blinded to experimental conditions whenever possible; readout of the neutralization assays was not performed with blinding, as these experiments often require subtle real-time adjustment to ensure optimal data collection.

Reporting for specific materials, systems and methods

We require information from authors about some types of materials, experimental systems and methods used in many studies. Here, indicate whether each material, system or method listed is relevant to your study. If you are not sure if a list item applies to your research, read the appropriate section before selecting a response.

Materials & experimental systems

n/a	Involved in the study	
☐	☒	Antibodies
☒	☐	Eukaryotic cell lines
☒	☐	Palaeontology and archaeology
☒	☐	Animals and other organisms
☒	☐	Human research participants
☒	☐	Clinical data
☐	☒	Dual use research of concern

Methods

n/a	Involved in the study	
☒	☐	ChIP-seq
☒	☐	Flow cytometry
☒	☐	MRI-based neuroimaging

Antibodies

Antibodies used

All antibodies used in the study were made recombinantly by cloning antibody heavy and light chains into the respective mammalian expression vectors. Antibodies were produced in mammalian cells (Expi293 cells) by transient transfection of expression vectors and purified by protein A affinity chromatography. The sequence, specificity, and function were verified for each antibody.

The influenza HA-targeting antibodies used in this work are listed in Table S1. These include:
22-1B08; 02-1D09; 04-1D10; 15-5E04; 55-1D06; 21-1A10 (this study)
315-19-1D12; 315-23-1C09; 315-55-1E08; and 315-55-1E11 (Creaga et al., Nat Commun 2021)
54-4H03; 58-6F03 (Wu et al., Cell Host & Microbe 2020)
310-33-1F04, 310-33-1G06 (Kanekiyo, et al., Nat Immunol. 2019)
315-02-1H01 (Corbett et al., mBio 2019)
315-02-1F07; 315-09-1B12; 315-27-1C08; 315-53-1A09; 315-53-1B06; 315-53-1F12; 13-1B02; 02-1B02 (Andrews et al., Sci Immunol 2017)
MEDI8852 (Kallewaard et al., Cell 2016)
CT149 (Wu et al., Nat Commun 2015)
F005-126 (Iba et al., J Virol 2014)
C05 (Ekiert et al., Nature 2012)

CR9114 (Dreyfus et al., Science 2012)
CR8020 (Ekiert et al., Science 2011)
FI6v3 (Corti et al., Science 2011)
CH65 (Whittle et al., PNAS 2011)
5J8 (Krause et al., J Virol 2011)
F045-092 (Ohshima et al., J Virol 2011)
CR6261 (Throsby et al., Plos One 2008)

Validation

Validation is described in detail in Nature Communications manuscript by Creanga et al. 2021 [https://doi.org/10.1038/s41467-021-21954-2]. All the antibodies used in the study were tested for their reactivity and specificity by ELISA, BLI using a set of recombinant HAs, or virus neutralization assays with multiple subtype viruses prior to use in the study.

Dual use research of concern

Policy information about dual use research of concern

Hazards

Could the accidental, deliberate or reckless misuse of agents or technologies generated in the work, or the application of information presented in the manuscript, pose a threat to:

No	Yes	
☒	☐	Public health
☒	☐	National security
☒	☐	Crops and/or livestock
☒	☐	Ecosystems
☒	☐	Any other significant area

Experiments of concern

Does the work involve any of these experiments of concern:

No	Yes	
☒	☐	Demonstrate how to render a vaccine ineffective
☒	☐	Confer resistance to therapeutically useful antibiotics or antiviral agents
☒	☐	Enhance the virulence of a pathogen or render a nonpathogen virulent
☒	☐	Increase transmissibility of a pathogen
☒	☐	Alter the host range of a pathogen
☒	☐	Enable evasion of diagnostic/detection modalities
☒	☐	Enable the weaponization of a biological agent or toxin
☒	☐	Any other potentially harmful combination of experiments and agents
